# Extracellular vesicles secreted from mesenchymal stem cells ameliorate renal ischemia reperfusion injury by delivering miR-100-5p targeting FKBP5/AKT axis

**DOI:** 10.1038/s41598-024-56950-1

**Published:** 2024-03-20

**Authors:** Guo Chen, Xinyuan Li, Xiang Zhou, Yang Li, Haitao Yu, Xiang Peng, Xuesong Bai, Chunlin Zhang, Zhenwei Feng, Yuhua Mei, Li Li, Yu Liu, Xin Gou, Yuanbin Jiang

**Affiliations:** 1 Department of Urology, The First Affiliated Hospital of Chongqing Medical University, Chongqing, 400000 China; 2 Department of Urology, Chongqing Traditional Chinese Medicine Hospital, No.6, Panxi Road(Branch7), Jiangbei, Chongqing, 400021 China; 3 Chongqing Key Laboratory of Molecular Oncology and Epigenetics, Chongqing, 400000 China

**Keywords:** Human umbilical cord mesenchymal stem cells, Small extracellular vesicles, miR-100-5p, FKBP5, Ischemia–reperfusion injury, Apoptosis, Biochemistry, Cell biology, Drug discovery, Physiology, Stem cells, Medical research, Molecular medicine, Nephrology, Pathogenesis, Urology

## Abstract

The incidence of acute kidney injury (AKI) due to ischemia–reperfusion (IR) injury is increasing. There is no effective treatment for AKI, and because of this clinical challenge, AKI often progresses to chronic kidney disease, which is closely associated with poor patient outcomes and high mortality rates. Small extracellular vesicles from human umbilical cord mesenchymal stem cells (hUCMSC-sEVs) play increasingly vital roles in protecting tissue function from the effects of various harmful stimuli owing to their specific biological features. In this study, we found that miR-100-5p was enriched in hUCMSC-sEVs, and miR-100-5p targeted FKBP5 and inhibited HK-2 cell apoptosis by activating the AKT pathway. HK-2 cells that were exposed to IR injury were cocultured with hUCMSC-sEVs, leading to an increase in miR-100-5p levels, a decrease in FKBP5 levels, and an increase in AKT phosphorylation at Ser 473 (AKT-473 phosphorylation). Notably, these effects were significantly reversed by transfecting hUCMSCs with an miR-100-5p inhibitor. Moreover, miR-100-5p targeted FKBP5, as confirmed by a dual luciferase reporter assay. In vivo, intravenous infusion of hUCMSC-sEVs into mice suffering from IR injury resulted in significant apoptosis inhibition, functional maintenance and renal histological protection, which in turn decreased FKBP5 expression levels. Overall, this study revealed an effect of hUCMSC-sEVs on inhibiting apoptosis; hUCMSC-sEVs reduced renal IR injury by delivering miR-100-5p to HK-2 cells, targeting FKBP5 and thereby promoting AKT-473 phosphorylation to activate the AKT pathway. This study provides novel insights into the role of hUCMSC-sEVs in the treatment of AKI.

## Introduction

Ischemia–reperfusion (IR) injury is one of the main causes of AKI; AKI is characterized by a sudden decrease in the glomerular filtration rate, as manifested by increased serum creatinine concentrations or oliguria, and AKI often leads to common and serious clinical complications, such as electrolyte disturbance, volume overload, drug toxicity and uremia^1,2^. In recent years, medical treatments have continuously improved, and the main methods that are currently used to treat AKI focus on prevention, active control of the primary etiology, treatment of complications, and drug treatments to ameliorate symptoms. In severe cases, patients undergo kidney replacement therapy^3^. Unfortunately, acute kidney injury is difficult to detect in early stages, and with currently available treatments, most patients still develop chronic kidney disease, with a mortality rate of 49.4%, even after kidney replacement therapy^4^. Therefore, it is still necessary to explore new therapeutic targets and accurate prognostic biomarkers to better understand the pathogenesis and pathophysiological process underlying renal IR injury.

Overwhelming evidence indicates that renal tubular epithelial cell (TEC) apoptosis is a key pathophysiological change that occurs during acute renal injury due to renal IR injury, and TEC apoptosis determines the degree of renal dysfunction^5,6^. Exploring additional valid regulatory approaches and targets by which to inhibit apoptosis is essential for the prevention and treatment of renal IR injury.

Mesenchymal stem cell therapy is a promising new direction for the treatment of kidney diseases, especially AKI^7^. Due to their capacities for tissue repair and regeneration, mesenchymal stem cells (MSCs), which are a diverse population of heterogeneous stromal cell subsets with self-renewal and multidirectional differentiation properties, have been shown to have clear therapeutic benefits on renal and liver ischemia–reperfusion (IR) injury^8,9^. For instance, Xie et al. demonstrated that MSCs accelerate the repair of IR-induced AKI by regulating macrophage polarization^10^. A study by Tseng et al. confirmed that MSCs ameliorate IR-induced AKI by downregulating the expression of the proinflammatory cytokine interleukin-1β, the proapoptotic protein Bax, and caspase 3 to enhance renal tubular autophagy^11^. According to Camila Eleuterio Rodrigues et al., MSCs inhibit the expression of aging-related genes in rats with AKI, reduce kidney fibrosis, and improve kidney function^12^. Jun Zheng et al. demonstrated that MSCs protect liver function during liver IR injury^13^. However, a number of risks, including high heterogeneity, uncertain differentiation, and poor survival of grafted MSCs, limit the efficacy of direct MSC transplantation into sites of injury. Moreover, paracrine approaches, such as the secretion of small extracellular vesicles (sEVs), have been confirmed to be the primary means by which MSCs exert their effects^14^. Consequently, interest in cell-free therapies that rely on sEVs derived from MSCs is increasing. With better plasticity, lower immunogenicity and higher levels of proliferation factors, sEVs derived from human umbilical cord MSCs (hUCMSC-sEVs) have shown substantial promise in the treatment of IR injury by regulating a variety of cellular processes, including apoptosis^15–17^. For instance, Li et al. demonstrated that sEVs from hUCMSCs protect against primary articular chondrocyte injury by delivering miR-100-5p^18^. A previous study showed that hUCMSC-sEVs can attenuate DOX-induced heart failure and protect cardiac function through the miR-100-5p/NOX4 pathway^19^. Moreover, Cao JY et al. quantified the miRNAs in hUCMSC‐sEVs and found that the content of miR-100-5p was the highest, approximately 16.13%, according to high-throughput miRNA sequencing (miRNA-seq)^20^. However, it remains unclear whether the protective effects of hUCMSC-sEVs against kidney IR injury occur due to the inhibition of apoptosis caused by miR-100-5p within these sEVs. Moreover, very few studies have directly revealed the therapeutic advantages and mechanism of hUCMSC-sEVs in AKI induced by renal IR injury, and even fewer studies have investigated the protective effect of hUCMSC-sEVs on apoptosis during renal IR injury.

In this study, we investigated the potential antiapoptotic effects of hUCMSC-sEVs against AKI due to IR injury in vivo and in vitro. We proved that renal tubular epithelial cell apoptosis was inhibited by hUCMSC-sEVs, resulting in a considerable reduction in kidney IR injury. Moreover, the underlying mechanism was revealed for the first time in our study. Specifically, renal tubular epithelial cell apoptosis was inhibited by hUCMSC-sEVs, at least in part via miR-100-5p/FKBP5/AKT signaling-mediated tubular repair. Our results clearly demonstrate that hUCMSC-sEVs are a novel treatment strategy for the restoration of renal function after AKI, providing new insights into the use of hUCMSC-sEVs in pertinent therapeutic approaches.

## Materials and methods

### Cell culture and treatment

Human renal cortex proximal convoluted tubule epithelial cells (HK-2 cells) and hUCMSCs were purchased from Pricella (Wuhan, China). Both types of cells were cultured in DMEM/F12 (Gibco, USA) supplemented with 12% fetal bovine serum (Gibco, USA) and 1% penicillin streptomycin in a 37 °C incubator with humidified air containing 5% CO_2_. To eliminate the interference of serum sEVs, the fetal bovine serum that was used to culture the hUCMSCs was ultracentrifuged at 120,000 g overnight^21^. To simulate the in vitro conditions of IR injury, we employed a chemical anoxia-recovery strategy. In particular, we established an oxygen and glucose deprivation and reoxygenation (OGD/R) model by culturing HK-2 cells for 1 h at 37 °C with 5% CO_2_ in a hypoxic solution comprising glucose-free media, antimycin A (5 mM), and 2oxy-D-glucose (5 mM), which is a glycolysis inhibitor^21^. Subsequently, instead of hypoxic medium, we immediately added complete DMEM/F12 medium and cultured the HK-2 cells for another 24 h. In vitro, we established a stem cell-HK-2 cell coculture model in which hUCMSCs were seeded on Transwell inserts (0.4 µm; Corning, NY). The inserts were then placed in six-well plates with HK-2 cells that were subjected to OGD/R conditions and cocultured for an additional twenty-four hours. This model was used to explore the protective effects of hUCMSC-derived paracrine sEVs on HK-2 cells during renal ischemia–reperfusion injury.

### Cell transfection

The negative control, miR-100-5p mimic sequence, and miR-100-5p inhibitor sequence were designed and acquired from Tsingke (Beijing, China). The siR-FKBP5 sequences were also designed and purchased from Tsingke. The Lipofectamine 2000 (Thermo Fisher, USA) reagent was used to transfect HK-2 cells with either the miR-100-5p mimic or inhibitor for 6–8 h. HK-2 cells were transfected with siR-FKBP5 by utilizing the Lipofectamine 3000 reagent (Thermo Fisher, USA) for 6–8 h. Subsequently, the cells were washed 2 times with PBS, and the cells from various transfection groups were grown in complete DMEM/F12 media for 24 h at 37 °C in an incubator with 5% CO_2_.

The sequences of the mimics, inhibitors and siRNAs that were used were as follows:

miR-100-5p mimics-F (sequence: 5′–AACCCGUAGAUCCGAACUUGUG–3′),

miR-100-5p mimics-R (sequence: 5′–CAAGUUCGGAUCUACGGGUUUU–3′),

miR-100-5p inhibitor (sequence: 5′–CACAAGUUCGGAUCUACGGGUU–3′),

siR-FKBP5 (sequence: 5′–GGGUAAACAGAUUGAGCAUdTdT–3′).

### Quantitative real‐time polymerase chain reaction

TRIzol reagent (Takara, Japan) was used to extract total RNA from cells or kidney tissues following the directions that were included in the RNA Extraction Kit. Reverse transcription of 1 µg of purified total RNA was performed with a PrimeScript RT reagent kit that included gDNA Eraser (Takara, Japan). With U6 and β-actin serving as internal controls, a SYBR (R) Prime-Script RT-PCR kit (Takara, Japan) was used to perform quantitative real-time PCR (qRT-PCR) with an ABI 7500 sequence detection system (Applied Biosystems, USA). The obtained ct values were normalized and statistically analyzed with GraphPad Prism 8.0. The primer sequences for miR-100-5p were as follows: forward, 5′–GCGAACCCGTAGATCCGAA–3′. The primer sequence for U6 was as follows: forward, 5′–CTCGCTTCGGCAGCACA–3′. The primer sequences for β-actin were as follows: forward, 5′–CCTTCCTGGGCATGGAGTC–3′; R, 5′–TGATCTTCATTGTGCTGGGTG–3′. The primer sequences for FKBP5 were as follows: forward: 5′–CAAGAAGTTTGCAGAGCAGGAT–3′; R: 5′–CACTGGGACTCTTCCCTCCTT–3′.

### sEV isolation and identification

Differential ultracentrifugation is thought to be the gold standard for EV separation^22^. hUCMSCs were cultured in culture medium supplemented with 12% FBS (with sEVs depleted). hUCMSC-sEV-rich supernatants were collected for sEV extraction by differential ultracentrifugation. After this step, non-sEV-conditioned medium (non‐sEV-CM) was isolated. The sEVs that were obtained by ultracentrifugation and dissolved in 200 µl of PBS were used for identification. The remaining sEVs were stored in a − 80 °C freezer.

We extracted hUCMSC-sEVs _(mimics)_ or hUCMSC-sEVs _(inhibitor)_ from hUCMSCs that were transfected with conditional medium containing miR-100-5p mimics or miR-100-5p inhibitor, respectively.

As the classic traditional method of identifying sEVs, tracking analysis technology (NTA) is used to comprehensively determine sEV particle size and concentration^23^. Transmission electron microscopy (TEM; Hitachi‐7500; Tokyo, Japan) was used to visualize the morphological structure of the sEVs. The expression levels of the sEV negative marker (CALNEXIN) and multiple sEV positive markers (CD9 and TSG101) were measured by Western blotting.

### sEV internalization

The separated sEVs were labeled in vitro for 4 min using the green fluorescent dye PKH67 (Sigma-Aldrich, MO), after which complete medium was added to prevent overstaining; then, ultracentrifugation was carried out for an additional 70 min at 120,000 × *g* and 4 °C. After washing with PBS, sEVs were added to HK‐2 cells and incubated for 24 h. PBS was used to wash the cells twice. ActinRed (Invitrogen, USA) was used to stain the cytoskeleton. The nuclei were counterstained with DAPI. In vivo, the sEVs were labeled with PKH26 (Sigma-Aldrich, MO) according to the same steps described above^21^. Laser scanning confocal microscopy was used to assess the internalization of sEVs both in vitro and in vivo.

### Flow cytometric analysis

To determine the proportion of cells undergoing apoptosis, HK-2 cells in various treatment groups were analyzed. After the cells were trypsinized for two minutes at 37 °C with 0.25% trypsin, they were centrifuged for 5 min at 1000 × *g*. After being harvested and resuspended in PBS, the HK-2 cells were stained with propidium iodide and Annexin V-FITC (Beyotime, China). Finally, the cells were examined using a flow cytometer (BD Biosciences, CA) to analyze the percentage of cells undergoing apoptosis.

### Western blotting analysis

After 15 min at 4 °C, MSC-sEVs, cells, and kidney tissues were lysed in a solution containing 1% PMSF and RIPA lysis buffer (Beyotime, China). The protein concentrations were measured according to the BCA assay (Thermo Fisher, USA) instructions. Equal quantities of protein samples were separated using 12% SDS-PAGE and then transferred to PVDF membranes (Millipore Sigma, USA). Considering proteins with similar molecular weight sizes, the pre-cut PVDF membranes with a width of 1.0 cm were prepared in advance to ensure that all PVDF bands were transferred and tested in the same Wb experiment. Gel electrophoresis and protein transfer were carried out according to standard procedures. Nonspecific binding was blocked by incubation with 5% nonfat milk for one hour at room temperature (RT), and the corresponding primary antibodies were then added and incubated overnight at 4 °C. The membranes were washed three times with Tris-buffered saline containing Tween (TBST). After a one-hour incubation with secondary antibodies at room temperature, the membranes were once again washed three times with TBST. Finally, we measured the levels of protein expression using electrochemiluminescence assays. The original WB data were showed in Supplementary information.

The primary antibodies that were used were as follows: anti-calnexin, anti-β-actin, anti-CD9, anti-TSG101, anti-Bax, anti-Bcl-2, anti-cleaved caspase-3, anti-FKBP5, anti-AKT, and anti-p-AKT antibodies. These antibodies were purchased from Proteintech, China.

### Luciferase reporter assay

The 293T cells were purchased from the Chinese Academy of Sciences Cell Bank (Shanghai, China). Using GP-transfect-Mate (GenePharma, China), 293T cells were cotransfected with Renilla luciferase, miRNA (miR-100-5p mimics or mimics-NC), and 3′UTR luciferase reporter constructs (pmirGLO, 3′UTR-FKBP5-WT, and 3′UTR-FKBP5-mutant). The luciferase activity of the cells was evaluated 48 h later using a microplate reader (Tecan M1000, Switzerland) and a Dual Luciferase Assay Kit (Promega, USA).

### In vivo kidney IR model

C57BL/6 male mice (20–25 g, 8–10 weeks old) from Chongqing Medical University's Animal Experiment Central were used to establish a bilateral kidney IR injury model. To assess the renoprotective effects of hUCMSC-sEVs on kidney IR injury, the mice were randomly assigned to 5 groups: (a) sham (n = 5); (b) IR + PBS (n = 5); (c) IR + hUCMSC-sEVs (n = 5); (d) IR + hUCMSC-sEVs _(mimics)_ (n = 5); and (e) IR + hUCMSC-sEVs_(inhibitor)_ (n = 5).

PBS (100 µl), hUCMSC-sEVs, hUCMSC-sEVs _(mimics)_ or hUCMSC-sEVs_(inhibitor)_ were administered via the tail vein (5 × 10^10^ particles in 100 µl of PBS) 6 h before IR injury.

The kidney IR injury model was established as follows. Pentobarbital sodium was intraperitoneally injected (75 mg/kg) to anesthetize the mice, and during surgery, the body temperatures of the animals were maintained between 36.5 and 37.5 °C. Surgical position: prone position. Surgical incision: Back incision (incision 1–1.5 cm along the back renal area). A non-traumatic vascular clamp was used to expose and obstruct the renal pedicles. Successful ischemia was indicated by an instantaneous change in kidney color from red to purple. The vascular clamp was removed 45 min later. The mice were ultimately sacrificed 24 h after reperfusion. In the sham group, the vasculature was not clamped during the operation. All blood samples were collected via apical punctures. Blood urea nitrogen (BUN) and serum creatinine (CREA) levels were measured using an automated biochemistry analyzer.

Every attempt was made to reduce the suffering of the animals. The animals were kept in a pathogen-free environment with a regulated 12-h light–dark cycle and free access to food and water. This study followed the American Veterinary Medical Association (AVMA) Guidelines for the Euthanasia of Animals (2020) as a comprehensive resource for guidance on veterinary best practices for the anesthesia and euthanasia of animals. The study involving live animals was reported as described by the ARRIVE guidelines (PLoS Bio 8(6), e1000412, 2010). The Chong Qing Medical University Animal Care and Use Committee approved this work, confirming that all the experiments were performed in accordance with relevant guidelines and regulations.

### Biochemical analysis

Blood samples were centrifuged (2000 × *g*, 15 min) to obtain serum. The Biochemical Laboratory of Sichuan Scientist Biotechnology Co., Ltd. (Sichuan, China) measured the amounts of BUN and CREA in the serum samples of each group, and five samples from each group were analyzed.

### HE staining biochemical analysis

Hematoxylin and eosin (HE) were used to evaluate renal histological morphology^24^. Kidneys from each group of mice were fixed in 4% formaldehyde, dehydrated in a graded series of ethanol solutions and xylene, and embedded in paraffin. To stain the slides (3 µm), hematoxylin and 0.5% eosin dye solution were used. Ultimately, the degree of renal tubular damage was determined based on tubular dilatation, cast formation, nuclear loss, and brush boundary loss. The degree of injury and correlative pathological scores were determined according to a previously described protocol^25^.

### Immunohistochemistry

Sections (3 µm) were cut from renal tissue samples that had been fixed in paraffin. FKBP5 and CC3 expression was detected using previously described methods^26^. After dewaxing, antigen repair, inactivation and normal goat serum blocking, the sections were treated with the corresponding primary antibodies and incubated overnight at 4 °C. The sections were first incubated for 30 min at 37 °C with secondary antibodies and then for another 30 min at 37 °C with a streptavidin–horseradish peroxidase conjugate. The sections were then stained with hematoxylin and DAB. Image-Pro Plus (IPP, v.6.0.206) was utilized for quantitative analysis to determine the expression levels of the corresponding markers.

### Statistical analysis

All the statistical analyses were performed with GraphPad Prism 8.0. All the results are reported as the mean ± standard deviation. To determine the statistical significance of differences between two groups, unpaired or paired 2-tailed Student's t tests were used. One-way ANOVA was applied for multigroup comparisons. A value of *p* < 0.05 was considered to indicate statistical significance.

## Results

### Identification and internalization of hUCMSC‐sEVs

**Figure 1:**
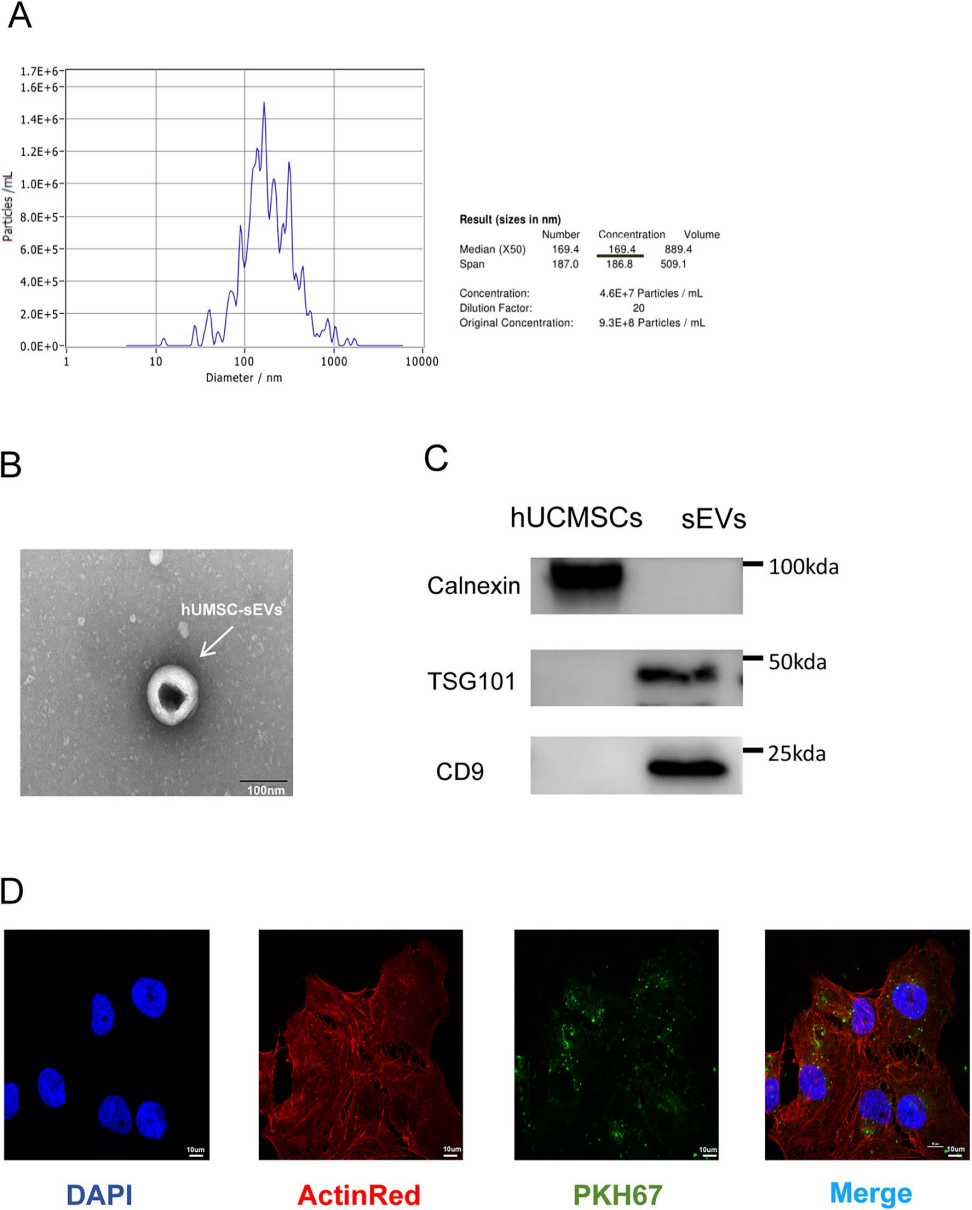
Characterization and internalization of hUCMSC-sEVs. (**A**) The sizes and concentrations of small extracellular vesicles from human umbilical cord mesenchymal stem cells (hUCMSC-sEVs) were determined using nanoparticle tracking analysis (NTA). (**B**) The typical morphology of hUCMSC-sEVs was observed by transmission electron microscopy (TEM, scale bar = 100 nm). (**C**) Western blotting analysis of CD9, TSG101 and calnexin expression in hUCMSCs and hUCMSC-sEVs. All PVDF bands are transferred and tested in the same Wb experiment. (**D**) Confocal microscopy images of PKH67-labeled hUCMSC-sEVs taken up by HK-2 cells (scale bar = 10 µm). (original magnification: 800 ×).

First, hUCMSC-sEVs were extracted from collected hUCMSC supernatants by ultrafast centrifugation. Then, sEVs in the extracted samples were identified in terms of morphology, size and common sEV surface markers. The NTA results revealed that the average diameter of the extracted samples was 169.4 nm, which was consistent with the size range of small cell-derived vesicles of 50–200 nm (Fig. 1A). Transmission electron microscopy (TEM) revealed typical particles with circular and double-coated clear membrane structures, consistent with the morphological characteristics of sEVs (Fig. 1B). To determine the relative expression of common sEV surface markers in the samples, we utilized Western blotting. The findings demonstrated that hUCMSC-sEVs expressed significant levels of sEV-associated markers (CD9 and Tsg101), while calnexin expression was not detected (Fig. 1C). We tagged hUCMSC-sEVs with PKH67 to verify that they were internalized by HK-2 cells. HK-2 cells were cultured with hUCMSC-sEVs for 24 h. The results showed that PKH67-labeled hUCMSC‐sEVs were internalized by both ActinRed- and DAPI-labeled HK‐2 cells (Fig. 1D).

### hUCMSC-sEVs inhibit OGD/R-induced HK-2 cell apoptosis

**Figure 2:**
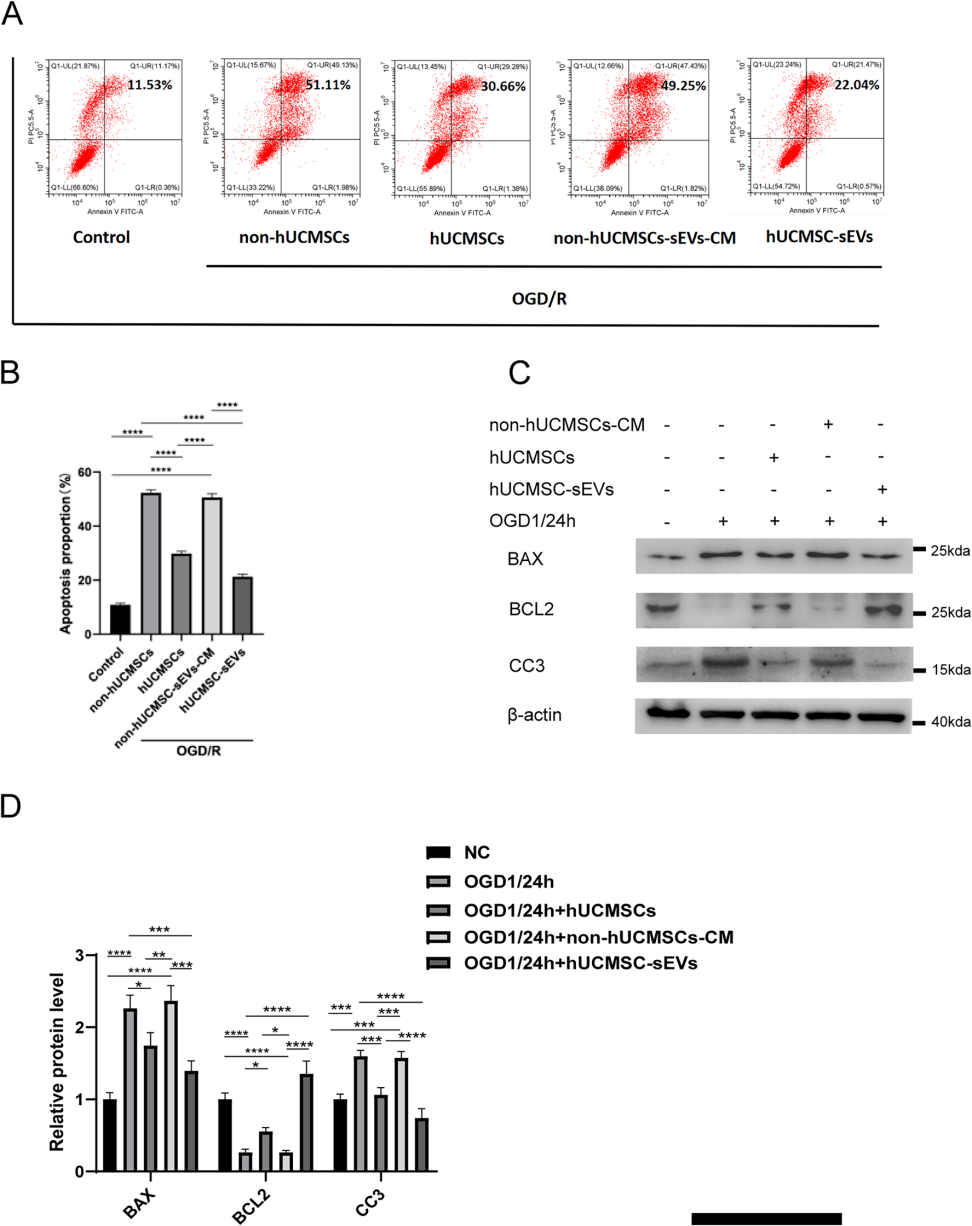
hUCMSC‐sEVs inhibit cell apoptosis during OGD injury. (**A**, **B**) The apoptosis of HK-2 cells subjected to various treatments was measured using flow cytometry. (**C**) Apoptosis-related protein (cleaved caspase-3, Bax, and Bcl-2) expression in HK-2 cells was measured by Western blotting. All PVDF bands are transferred and tested in the same Wb experiment. (**D**) Statistical analysis of apoptosis-related protein levels in HK-2 cells.

One recent study showed that renal ischemia for 1 h and reperfusion for 24 h leads to significant renal IR injury^27^. Based on previous studies, we determined that the time point for investigating renal IR injury should be after 1 h of ischemia and then 24 h of reperfusion. To investigate whether hUCMSC-sEVs can exert a protective effect on OGD/R-induced AKI by preventing HK-2 cell apoptosis, flow cytometry was utilized to determine the percentage of apoptotic HK-2 cells after exposure to various treatments. Compared with the control condition, OGD/R considerably promoted HK-2 cell apoptosis. Interestingly, coculture with hUCMSCs or the addition of hUCMSC-sEVs significantly reversed this increase in apoptosis (Fig. 2A,B). Moreover, we explored whether hUCMSC-sEVs can regulate the expression of proteins that are associated HK-2 cell apoptosis after OGD/R injury. Western blotting was used to measure the expression of classical apoptosis-associated proteins (cleaved caspase-3, Bax, and Bcl-2) in each group. OGD/R-treated cells exhibited significantly decreased Bcl‐2 expression and markedly increased Bax and cleaved caspase‐3 expression. Coculture with hUCMSCs or the addition of hUCMSC-sEVs significantly reversed these changes in protein expression (Fig. 2C,D).

In addition, we found that coculture with hUCMSCs or treatment with hUCMSC-sEVs had significant inhibitory effects on OGD/R-mediated apoptosis, but these effects were not observed in the group treated with hUCMSC conditioned medium without sEVs; these results suggested that the sEVs of hUCMSCs mediated the protective effects of the hUCMSCs (Fig. 2A,C). These results suggest that sEVs secreted by hUCMSCs are essential for protecting against OGD/R injury by inhibiting HK-2 cell apoptosis during renal IR injury.

### miR‐100‐5p is enriched in hUCMSC‐sEVs and is associated with the protective effects of hUCMSC‐sEVs on kidneys

**Figure 3:**
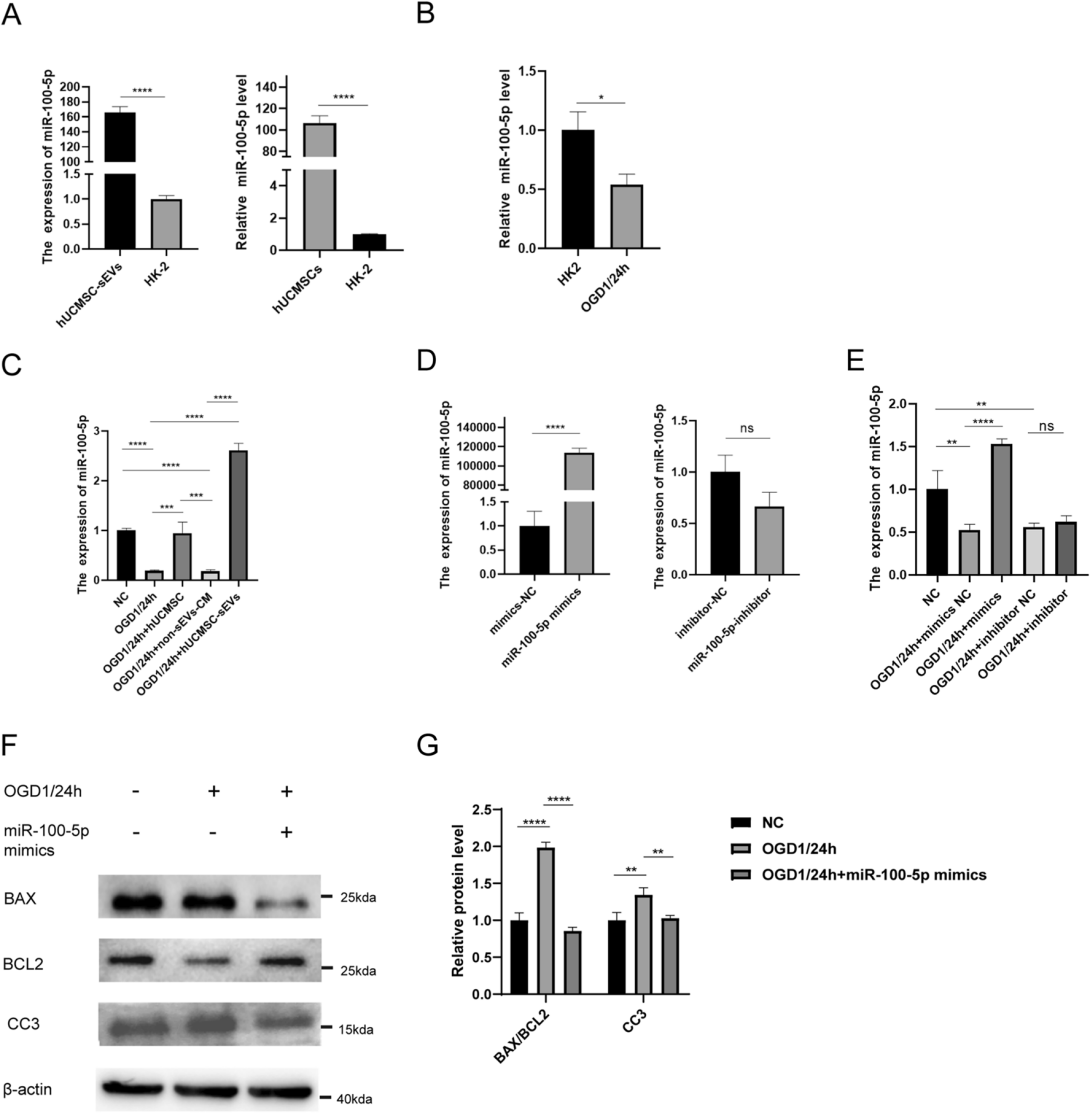
hUCMSC-sEVs are enriched in miR-100-5p and deliver it to HK-2 cells, thereby inhibiting HK-2 cell apoptosis induced by OGD/R injury. (**A**) The expression levels of miR-100-5p were measured by qPCR. (**B**) miR-100-5p levels in cells before and after OGD1/24 h treatment as determined by qPCR. (**C**) Transcription level of miR-100-5p in each group. (**D**) Detection of miR-100-5p levels by qPCR after HK-2 cells were transfected with the miR-100-5p inhibitor or mimic. (**E**) Detection of miR-100-5p expression levels by qPCR after OGD/R-exposed HK-2 cells were transfected with the miR-100-5p inhibitor or mimic. (**F**) Effect of miR-100-5p overexpression on the levels of apoptosis-related proteins in HK-2 cells subjected to OGD/R. All PVDF bands are transferred and tested in the same Wb experiment. (**G**) Statistical analysis of the levels of apoptosis-related proteins in HK-2 cells subjected to different treatments.

Previous studies revealed that according to miRNA-seq analysis of hUCMSC-sEVs, among all the miRNAs that are carried by hUCMSC-sEVs, miR-100-5p is the most abundant in hUCMSC-sEVs^18^. Since miR-100-5p has not been studied in kidney ischemia–reperfusion, we chose miR-100-5p, which is highly expressed in hUCMSC-sEVs, as the research object to explore the mechanism underlying its effects on renal ischemia–reperfusion injury. First, to verify whether hUCMSC-sEVs contain high levels of miR-100-5p, we extracted RNA from hUCMSC-sEVs and hUCMSCs and then conducted qPCR experiments. The findings demonstrated that miR-100-5p levels in hUCMSCs and hUCMSC-sEVs was much greater than that in normal HK-2 cells (Fig. 3A). Moreover, the levels of miR-100-5p in HK-2 cells was further decreased in the OGD/R model group (Fig. 3B). This indicates that the addition of exogenous HUCMSC-sEVs or coculture with hUCMSCs can result in the delivery of miR-100-5p to damaged HK-2 cells during renal IR injury via paracrine sEVs. Moreover, the qPCR results revealed that the expression of miR-100-5p was upregulated by coculture with hUCMSCs or treatment with hUCMSC-sEVs, but this effect was not observed in the group treated with hUCMSC conditioned medium without sEVs (Fig. 3C). These results raised the possibility that miR-100-5p plays a role in kidney IR injury. To further clarify the specific mechanism by which miR-100-5p functions in renal ischemia–reperfusion injury, we first established miR-100-5p knockdown and overexpression models by transfecting mimics or inhibitors into HK-2 cells. The findings showed that miR-100-5p expression was significantly upregulated in HK-2 cells after transfection with miR-100-5p mimics but was not significantly downregulated in HK-2 cells after transfection with the miR-100-5p inhibitor (Fig. 3D). This result may have occurred due to the low background expression of miR-100-5p in HK-2 cells, such that after transfection of the miR-100-5p inhibitor into HK-2 cells, the downregulation of miR-100-5p expression was not significant. Next, we explored the effects of the miR-100-5p mimic or inhibitor on the expression level of miR-100-5p in HK-2 cells subjected to OGD/R. Notably, in renal OGD/R injury, the mimic significantly increased the levels of miR-100-5p, while inhibitor did not significantly reduce the expression level of miR-100-5p (Fig. 3E). Therefore, considering that the miR-100-5p expression level in HK-2 cells is low and that the miR-100-5p expression level is further downregulated in HK-2 cells after OGD/R, we used a mimic overexpression model instead of an inhibitor knockdown model in the subsequent investigation of the function of miR-100-5p and underlying mechanism. We further explored the function of miR‐100‐5p during OGD/R injury. HK-2 cells were transfected with the miR-100-5p mimic and immediately subjected to OGD/R. Compared with the OGD/R group, the OGD/R + miR-100-5p mimic group exhibited markedly downregulated expression of HK-2 apoptosis-related proteins (cleaved caspase-3 and BAX/BCL2) after OGD/R injury (Fig. 3F,G). Consequently, miR-100-5p in hUCMSC-sEVs was essential for the hUCMSC-sEV-mediated inhibition of OGD/R injury-induced apoptosis.

### miR-100-5p inhibits FKBP5 expression

**Figure 4:**
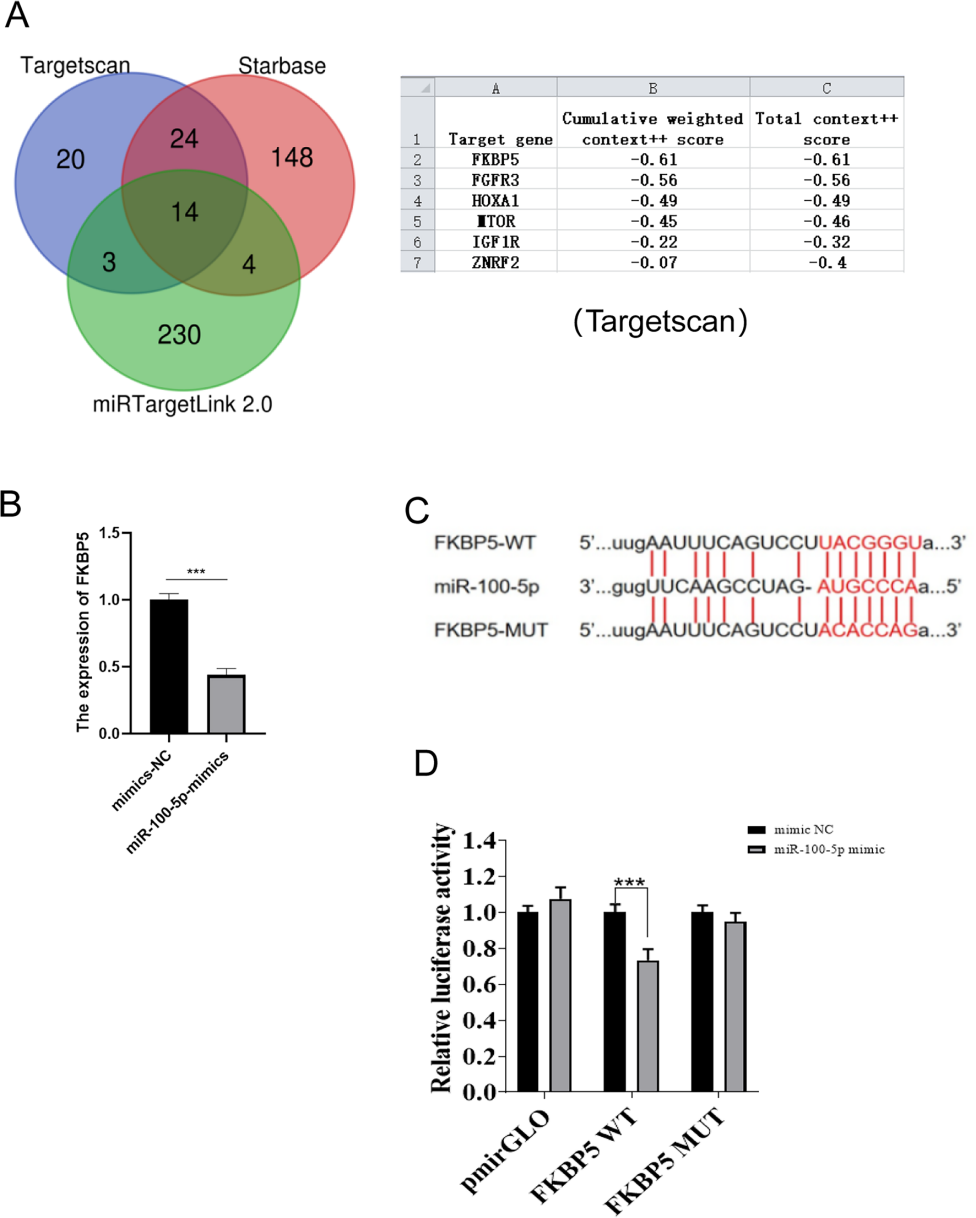
Potential targets of miR-100-5p. (**A**) Venn diagram illustrating the genes associated with miR-100-5p. Six key target genes associated with ischemia and reperfusion that were predicted by these three websites were identified. (**B**) Detection of FKBP5 mRNA expression levels after miR-100-5p mimic transfection by qPCR. (**C**) Schematic diagram of the predicted binding sites of miR-100-5p to the FKBP5 mRNA 3'UTR and mutant FKBP5 mRNA 3'UTR by TargetScan. (**D**) FKBP5 was confirmed to be a miR-100-5p target by a luciferase reporter assay.

To further elucidate the potential mechanism by which miR-100-5p exerts its effects, three online databases—TargetScan, Starbase, and miRTargetLink 2.0—were used to predict the probable targets of miR-100-5p that may be involved in the regulation of cell apoptosis. By overlapping the predicted results of these three sites, we identified a total of 14 possible target genes. There were 6 genes related to ischemia and reperfusion, namely, MTOR, HOXA1, FKBP5, ZNRF2, IGF1R and FGFR3. We further queried the cumulative weighted scores and total scores of miR-100-5p on the above genes on the TargetScan website, and FKBP5 ranked first (Fig. 4A). Therefore, we explored whether miR-100-5p regulates FKBP5 expression. First, HK-2 cells were transfected with the miR-100-5p mimic to establish a miR-100-5p overexpression model. At the transcription level, compared with the mimic NC group, the HK-2 cells in the miR-100-5p mimic group exhibited significantly reduced FKBP5 expression. This indicates the presence of a regulatory relationship between miR-100-5p and FKBP5 (Fig. 4B). Notably, the luciferase reporter assay showed that compared to miRNA NC, miR-100-5p overexpression significantly decreased the activity of luciferase reporters. Moreover, the miR-100-5p overexpression vector (pmirGLO) had no effect on the activity of the FKBP5-3′-UTR-mutant luciferase reporter, indicating that FKBP5 was a bona fide target of miR-100-5p (Fig. 4C,D).

### hUCMSC-sEVs maintain phospho-AKT (Ser473) phosphorylation and decrease apoptosis by inhibiting FKBP5 signaling via miR-100-5p

**Figure 5:**
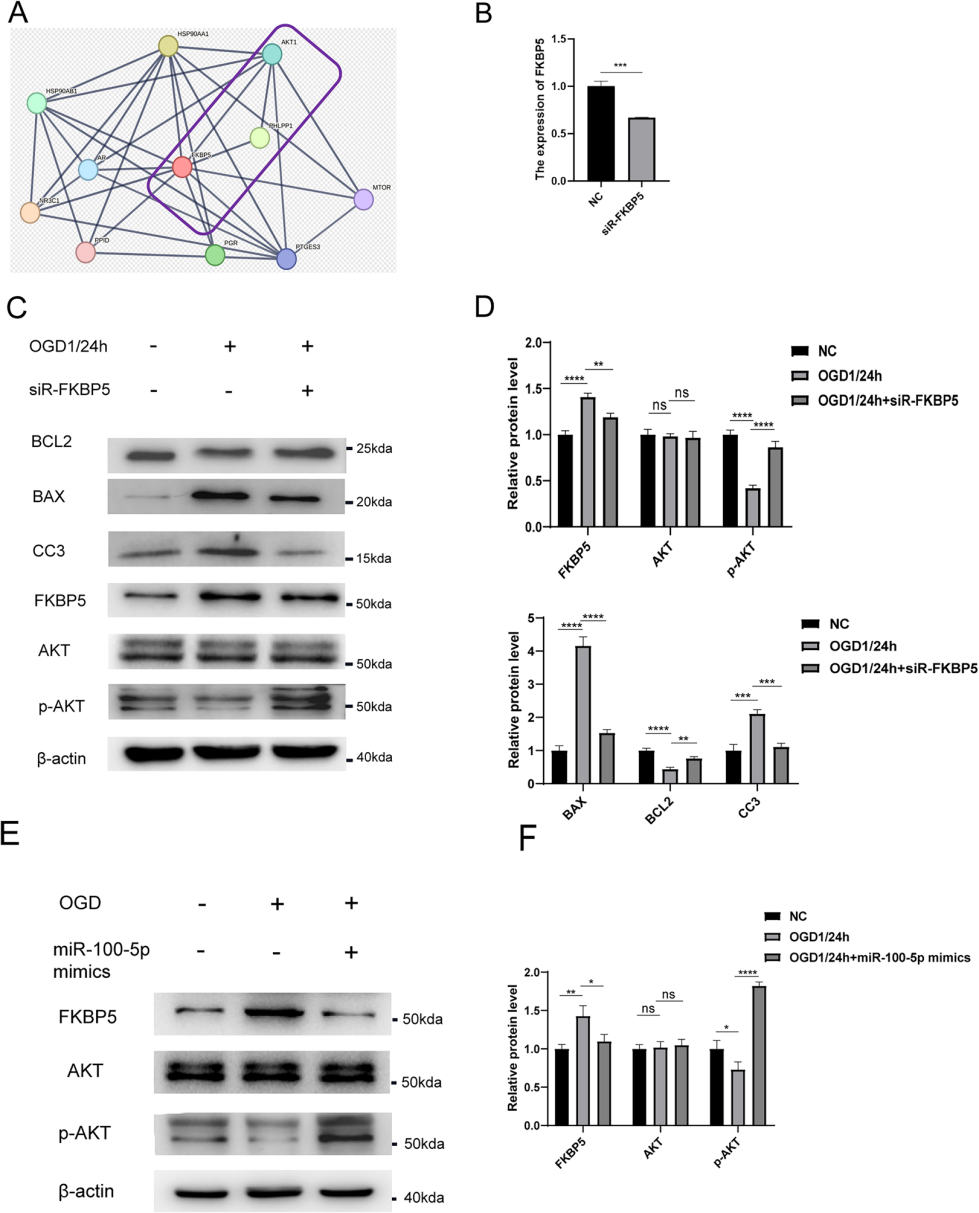

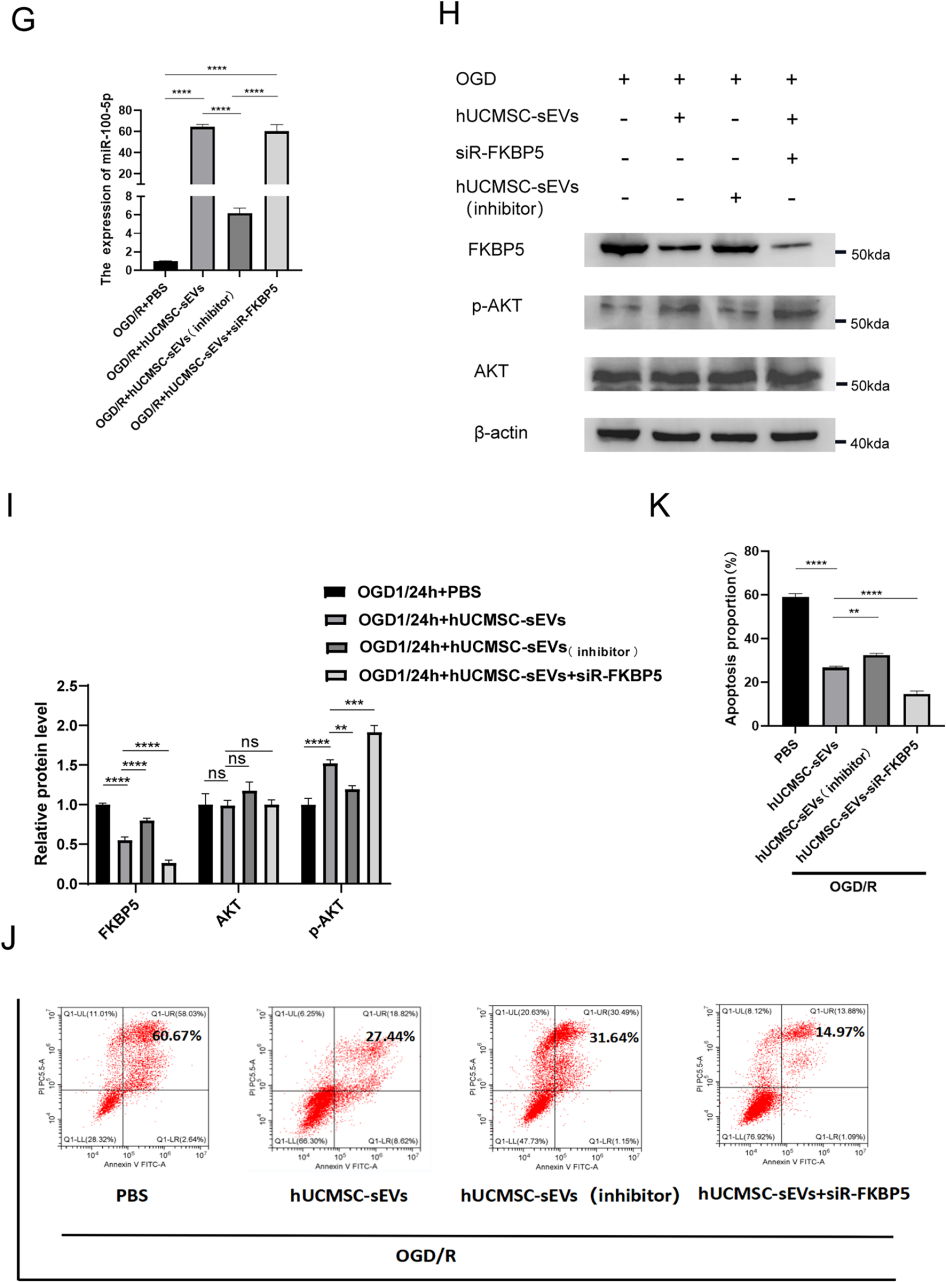
miR-100-5p within hUCMSC-sEVs targets FKBP5, increases AKT Ser473 phosphorylation and subsequently activates the AKT pathway. (**A**) Protein interaction correlation analysis of FKBP5 predicted by the String online website. (**B**) Measurement of FKBP5 mRNA expression levels in HK-2 cells transfected with siR-FKBP5 or the control by qPCR. (**C**) HK-2 cells were transfected with siR-FKBP5 and then subjected to OGD/R, and the levels of AKT Ser473 phosphorylation-related and apoptosis-related proteins were measured by Western blotting. All PVDF bands are transferred and tested in the same Wb experiment. (**D**) Statistical analysis of the levels of AKT Ser473 phosphorylation pathway-related and apoptosis-related proteins in HK-2 cells subjected to different treatments. (**E**) Western blotting was used to measure the levels of AKT Ser473 phosphorylation pathway-related proteins. All PVDF bands are transferred and tested in the same Wb experiment. (**F**) Statistical analysis of the levels of AKT Ser473 phosphorylation pathway-related proteins in HK-2 cells subjected to different treatments. (**G**) The levels of miR-100-5p in HK-2 cells transfected with siR-FKBP5 and cultured with hUCMSC-sEV- or hUCMSC-sEV _(inhibitor)_-conditioned medium. (**H**) The levels of AKT Ser473 phosphorylation pathway-related proteins in HK-2 cells subjected to different treatments were detected by Western blotting. All PVDF bands are transferred and tested in the same Wb experiment. (**I**) Statistical analysis of the levels of AKT Ser473 phosphorylation pathway-related proteins in HK-2 cells subjected to different treatments. (**J**) The apoptosis of HK-2 cells transfected with or without siR-FKBP5 and treated with different agents was determined via flow cytometry. (**K**) Statistical analysis of HK-2 cell apoptosis.

Liu BC et al. revealed that FKBP51 can play a crucial role in body metabolism, tumorigenesis and drug resistance via the AKT pathway^28^. Moreover, using the String Study Protein Interaction Prediction website (https://cn.string-db.org/), we predicted the networks of proteins that interact with FKBP5. FKBP5 is most closely associated with the phosphorylation of AKT (Ser473) and PHLPP^29^ (Fig. 5A). Interestingly, Pei H et al. showed that the phosphatase PHLPP specifically dephosphorylates the hydrophobic motif of AKT (Ser473 in AKT), thereby negatively regulating AKT activity, and FKBP5 acts as a scaffold protein between AKT and PHLPP, promoting the dephosphorylation of AKT by PHLPP and thereby reducing AKT activity^29^. Therefore, we hypothesized that hUCMSC-sEVs inhibit the FKBP5 signaling pathway by delivering miR-100-5p to HK-2 cells, maintaining phospho-AKT (Ser473) phosphorylation, and reducing apoptosis during renal ischemia–reperfusion. We first transfected siR-FKBP5 into HK-2 cells and successfully established an FKBP5-knockdown model (Fig. 5B).

We investigated whether miR-100-5p and FKBP5 can inhibit HK-2 cell apoptosis during kidney IR injury via the AKT (Ser473) phosphorylation pathway. Western blotting analysis revealed that compared with the OGD/R group, the OGD/R + miR-100-5p mimic group and OGD/R + siR-FKBP5 group had distinctly reduced FKBP5 expression and increased AKT phosphorylation (Ser473), which further inhibited HK-2 cell apoptosis during renal IR injury; moreover, the expression of cleaved caspase-3 and BAX was markedly downregulated, and the expression of BCL2 was upregulated (Figs. 3E and 5C–F).

Therefore, we hypothesized that miR‐100‐5p in hUCMSC-sEVs regulated FKBP5 and activated the AKT pathway to prevent HK-2 cell apoptosis during renal ischemia–reperfusion injury. To verify our hypothesis, we used qPCR to measure the levels of miR-100-5p in HK-2 cells subjected to various treatments. We transfected miR-100-5p into hUCMSCs, collected hUCMSC-conditioned medium, extracted small extracellular vesicles, and obtained hUCMSC-sEVs _(inhibitor)_. We found that the addition of hUCMSC-sEVs to HK-2 cells that were exposed to OGD/R significantly increased the levels of miR-100-5p, while the addition of hUCMSC-sEVs _(inhibitor)_ greatly reduced the increase in the miR-100-5p levels (Fig. 5G). Western blotting analysis further revealed that the inhibitory effect of OGD/R + hUCMSC-sEVs _(inhibitor)_ on FKBP5 was markedly inhibited compared with that of OGD/R + hUCMSC-sEVs. Moreover, the inhibitory effect on AKT phosphorylation (at Ser473) was markedly increased. There was no significant change in the regulation of AKT among the different groups (Fig. 5H,I). Interestingly, by flow cytometry, we found that the inhibitory effect of HK-2 cell apoptosis on renal ischemia–reperfusion injury was dramatically reduced in the OGD/R + hUCMSC-sEV _(inhibitor)_ group compared with that in the OGD/R + hUCMSC-sEV group (Fig. 5J,K). Notably, we also investigated the function of FKBP5 in the hUCMSC-sEV model of renal ischemia–reperfusion injury. Similarly, flow cytometry and Western blotting showed that the expression of FKBP5 was further downregulated in the OGD/R + hUCMSC-sEVs + siR-FKBP5 group, and the level of phosphorylated AKT (Ser473) was markedly increased, which inhibited HK-2 cell apoptosis during kidney IR injury and protected renal function, compared with those in the OGD/R + HUCMSC-sEV group (Fig. 5H–K). The results revealed that miR-100-5p in hUCMSC-sEVs can inhibit HK-2 cell apoptosis during kidney IR injury by targeting FKBP5 and maintaining AKT phosphorylation (Ser473).

### hUCMSC-sEVs mitigate renal IR injury in vivo by delivering miR-100-5p to target FKBP5

**Figure 6:**
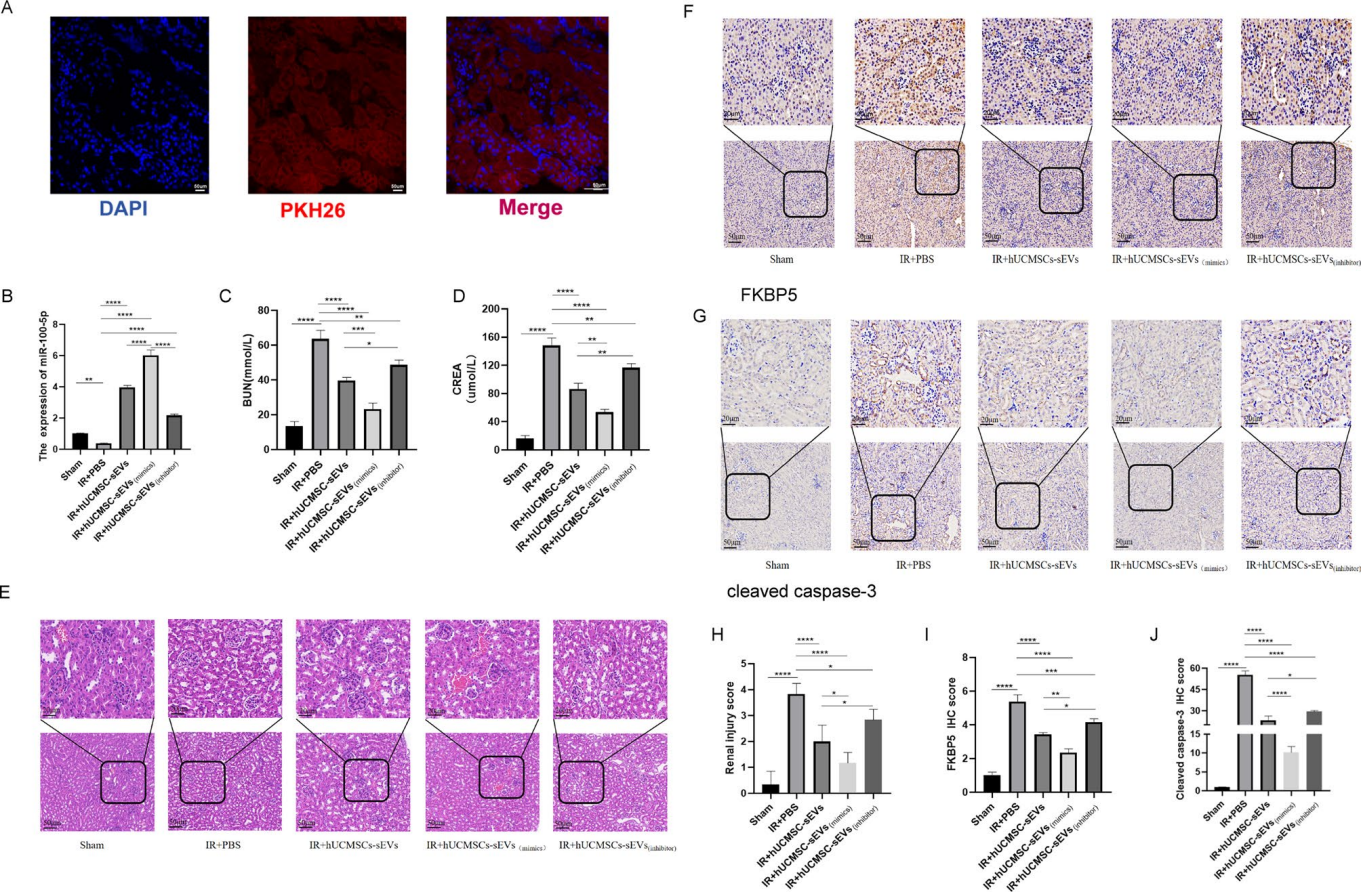
hUCMSC-sEVs ameliorate IR injury by downregulating FKBP5 and inhibiting apoptosis. (**A**) hUCMSC-sEVs labeled with PKH26 were absorbed into kidney tissues after ischemia for 1 h and reperfusion injury for 24 h. Scale bar = 50 μm (original magnification: 600 ×). (**B**) The levels of miR-100-5p in kidney tissues subjected to IR injury or control and treated with hUCMSC-sEVs, hUCMSC-sEVs (inhibitors), or hUCMSC-sEVs _(mimics)_ were measured. (**C**, **D**) hUCMSC-sEVs, hUCMSC-sEVs _(mimics)_, and hUCMSC-sEVs_(inhibitor)_ ameliorated the renal IR injury-induced increases in CREA and BUN levels to different degrees. (**E**) Representative HE staining images of kidney tissue sections from different treatment groups (scale bar = 50 µm; 20 µm). (**F**, **G**) Immunohistochemical staining showing the expression of FKBP5 and cleaved caspase-3 in the different treatment groups (scale bar = 50 µm; 20 µm). (**H**) Renal injury score in different treatment groups based on nuclear loss, tubular dilation, cast formation, and brush border loss. (**I**, **J**) FKBP5 and cleaved caspase-3 immunohistochemical scores of the different treatment groups.

To adequately evaluate and validate the function of hUCMSC-sEVs and miR-100-5p within these sEVs during renal IR injury in vivo, we injected PBS, hUCMSC-sEVs, hUCMSC-sEVs _(mimics)_ or hUCMSC-sEVs_(inhibitor)_ via the tail vein and established a mouse renal ischemia–reperfusion injury model by clamping both renal pedicles. First, we labeled hUCMSC-sEVs with PKH26 and discovered that a large number of hUCMSC-sEVs accumulated in the renal tissues of the mice (Fig. 6A). We extracted RNA from kidney tissue from each group and subsequently observed that the expression level of miR-100-5p in the IR + hUCMSC-sEVs (inhibitor) group was significantly lower than that in the IR + hUCMSC-sEVs group. Moreover, we found that hUCMSC-sEVs _(mimics)_ further increased the expression level of miR-100-5p in hUCMSC-sEVs (Fig. 6B). Supplementation with exogenous hUCMSC-sEVs, hUCMSC-sEVs _(mimics)_ or hUCMSC-sEVs_(inhibitor)_ attenuated bilateral kidney IR injury to different degrees in terms of function and morphology, as indicated by the SCr and BUN concentrations (Fig. 6C,D) and H&E staining results (Fig. 6E,H). In renal IR injury, the protective effect of exogenous hUCMSC-sEV supplementation on renal function was significantly greater than that of exogenous hUCMSC-sEV _(inhibitor)_ supplementation, but it was significantly less pronounced than that of hUCMSC-sEV_(mimics)_ supplementation. Moreover, IHC experiments were performed to detect FKBP5 and cleaved caspase-3 expression levels in vivo. After bilateral renal IR treatment, the expression of FKBP5 and cleaved caspase-3 was significantly upregulated. Exogenous hUCMSC-sEV_(mimics)_ supplementation improved the upregulation of these molecules via the targeting of FKBP5 by miR-100-5p to a greater extent than did exogenous hUCMSC-sEV supplementation (Fig. 6F,G,I,J). These data were consistent with the in vitro results, providing compelling evidence that hUCMSCs have a protective effect on renal IR injury, and this effect is closely related to the expression level of miR-100-5p in hUCMSCs. Overall, hUCMSC-sEVs inhibit apoptosis, mitigate kidney IR injury and protect renal function during renal ischemia–reperfusion injury by delivering miR-100-5p, which targets FKBP5.

## Discussion

Ischemia–reperfusion injury is the main cause of AKI and is associated with a high mortality rate. There are still many gaps in our understanding of renal IR injury, including our understanding of early prediction methods and treatment strategies. Relevant research has shown that the primary tubule fragments that suffer ischemic damage during AKI are in the proximal tubular epithelium^18^. Because the proximal tubules are densely packed with mitochondria, tubular epithelial cells exhibit active metabolic activity and are highly sensitive to ischemic stimuli and hypoxia^28^. Under stress conditions, changes in mitochondrial permeability and fragmentation, which lead to mitochondrial pathway-mediated tubular epithelial cell apoptosis, are the most common pathological changes that occur during AKI^28–31^. Clearly, inhibition of the mitochondrial apoptosis pathway that is associated with activated caspase-3 and BAX/BCL2 can significantly inhibit AKI progression and promote renal function repair^9,31^.

It has been confirmed that hUCMSCs, which are recently discovered stem cells with extensive self-renewal and pluripotent differentiation abilities, are involved in the repair of different organs after IR injury mainly via sEVs that are secreted by paracrine pathways^32–34^. hUCMSC-sEVs, which are carriers of biomolecules that are related to intercellular communication, are safer and deliver specific miRNAs to perform their biological functions in target cells, such as the regulation of angiogenesis, fibrosis and proliferation^35–38^. For instance, a previous study confirmed that extracellular vesicles that are secreted by mesenchymal stem cells ameliorate kidney IR injury by inhibiting mitochondrial fission via miR-30^39^. Zhang et al. demonstrated that extracellular vesicles that are secreted by adipose-derived mesenchymal stem cells improve cerebral ischemic injury by delivering miR-22-3p, which targets the KDM6B/BMP2/BMF axis^40^. Ou et al. revealed that extracellular vesicles that are secreted by mesenchymal stem cells protect the heart against IR injury by delivering miR-150-5p^41^. Our study demonstrated for the first time that miR-100-5p is abundant in both hUCMSCs and hUCMSC-sEVs, and it can be absorbed by renal TECs, inhibiting the mitochondrial pathway-mediated apoptosis of renal TECs via the delivery of miR-100-5p and thereby protecting renal function during renal ischemia–reperfusion injury. hUCMSC-sEVs may be a promising new direction for the treatment of AKI. The following results support this conclusion: (1) The expression level of miR-100-5p in HK-2 cells that are exposed to IR injury was further downregulated, and as a therapeutic approach, the delivery of exogenous sEVs secreted by hUCMSCs to HK-2 cells that were exposed to IR injury significantly increased the level of miR-100-5p and inhibited IR injury-induced apoptosis. (2) HK-2 cells that were exposed to IR injury and cocultured with hUCMSC-derived conditioned medium or sEVs exhibited increased miR-100-5p levels and inhibited renal cell apoptosis during kidney IR injury. (3) In a mouse renal IR injury model, by using hUCMSC-sEVs with different miR-100-5p levels, we demonstrated that hUCMSC-sEV therapy significantly inhibited mitochondrial pathway-mediated apoptosis by delivering miR-100-5p, thereby attenuating renal tubular injury.

To further elucidate the potential mechanism by which miR-100-5p inhibits apoptosis in the IR injury model, we first identified the target gene of miR-100-5p by utilizing TargetScan, starBase, and miRTargetLink 2.0 software; the results predicted that FKBP5 might be the specific target gene of miR-100-5p. In our study, a negative correlation between miR-100-5p levels and FKBP5 levels was detected for the first time, and a luciferase reporter confirmed the presence of RNA interactions. FKBP5 belongs to a class of immunophilin proteins and is involved mainly in protein folding, protein transport, and immune regulation^42,43^. Some reports have also revealed a role for FKBP5 in promoting apoptosis and autophagy^44^. A recent study indicated that the inhibition of FKBP5 expression and the promotion of AKT phosphorylation are essential for protecting neurons from apoptosis induced by OGD/R injury^45^. Luan P et al. demonstrated that the targeting of FKBP5 by miR-9-5p inhibits the apoptosis of renal lymphocytes via the mitochondrial pathway^46^. These findings raise the possibility that FKBP5 may be involved in TEC apoptosis during renal IR injury. Our study fully demonstrated that the increase in FKBP5 expression during renal IR injury was closely related to renal IR injury-induced apoptosis because this effect disappeared in HK-2 cells that were subjected to kidney IR injury and transfected with siR-FKBP5. In renal IR injury, exogenous supplementation with hUCMSC-sEVs significantly increased the level of miR-100-5p, which targets FKBP5, in HK-2 cells, thereby inhibiting apoptosis via the mitochondrial pathway.

Emerging findings prove that the AKT1 signaling pathway and the level of AKT1 phosphorylation at Ser 473 (P-AKT-473) are related to the signaling pathway that controls cell survival, death and proliferation^47^. Previous studies have shown that under conditions of growth factor stimulation, Ser473 in the AKT1 regulatory domain can be phosphorylated to regulate apoptosis-related proteins, such as Bax, Bcl2, cleaved caspase-3, and Caspase-9^48,49^. A recent study indicated that FKBP5 acts as a scaffold protein between AKT and PHLPP, and PHLPP has been shown to selectively dephosphorylate the hydrophobic motif of AKT (Ser473 in AKT), inhibiting the AKT signaling pathway^29,50^. Miyamoto et al. demonstrated a positive correlation between the level of AKT-473 dephosphorylation and cardiomyocyte apoptosis^50^. As recently reported, the FKBP5/AKT pathway also regulates mitochondrial apoptosis pathways in lymphocytes^47^. The FKBP5-AKT pathway has been proven to be involved in cerebral ischemic stroke^46^. However, whether FKBP5 also modulates cell apoptosis via the dephosphorylation of AKT-473 during kidney IR injury is unclear. In this study, in the hUCMSC and hUCMSC-sEV groups, FKBP5 was suppressed; this simultaneously increased AKT phosphorylation at Ser473, which was accompanied by mitochondrial apoptosis pathway inhibition. By coculturing hUCMSC-sEVs containing different miR-100-5p levels with HK-2 cells that were exposed to kidney IR injury, our study further confirmed that miR-100-5p within hUCMSC-sEVs suppressed epithelial cell apoptosis by targeting FKBP5-AKT signaling to promote IR injury repair in the kidney.

In this study, we administered hUCMSC-sEVs carrying different miR-100-5p concentrations to mice via tail vein injection before IR injury and subsequently evaluated the ability of hUCMSC-sEVs to restore renal tubule function after IR injury. We found that after hUCMSC-sEV treatment, the expression of miR-100-5p was increased, the expression of FKBP5 and proteins related to mitochondrial apoptosis was decreased, the levels of CREA and BUN were decreased, and renal morphology and function were ameliorated. Most significantly, we revealed a novel mechanism underlying hUCMSC function: hUCMSC‐sEVs deliver miR‐100‐5p to renal tubular epithelial cells and inhibit apoptosis by decreasing FKBP5 expression and subsequently activating the AKT signaling axis.

Some limitations in our present study should be considered. The possibility that other miR-100-5p targets, such as BAX, may also produce marked effects cannot be completely excluded. Moreover, although we demonstrated that renal tubular epithelial cells can take up hUCMSC-sEVs both in vivo and in vitro, we were unable to determine the specific mouse organ where hUCMSC-sEVs accumulate because of insufficient research conditions, so this topic will be further explored.

## Conclusion

Our findings suggest that miR-100-5p within hUCMSC-sEVs inhibits renal tubular epithelial cell apoptosis during renal IR injury. More importantly, the underlying mechanisms were revealed to involve the regulation of FKBP5 by miR-100-5p and the subsequent phosphorylation of AKT-473. This study reveals a novel mechanism underlying AKI treatment with hUCMSC‐sEVs and provides new ideas and therapeutic strategies for protecting kidney function during the progression of AKI to CKD.

### Supplementary Information

Supplementary Information.

## Data Availability

All data supporting the results reported here are available in the article or from the corresponding author upon reasonable request.
