# Supplementary material for: Extracellular vesicles secreted from mesenchymal stem cells ameliorate renal ischemia reperfusion injury by delivering miR-100-5p targeting FKBP5/AKT axis
Source: Sci Rep. 2024 Mar 20;14:6720. doi: 10.1038/s41598-024-56950-1 (PMC10954733; doi:10.1038/s41598-024-56950-1)

SPRINGER NATURE

Author Services

Editing Certificate

This document certifies that the manuscript

Extracellular vesicles secreted from mesenchymal stem cells improve renal ischemia-reperfusion injury by delivering miR-100-5p targeting FKBP5/AKT axis

prepared by the authors

Guo Chen，Yuanbin Jiang

was edited for proper English language, grammar, punctuation, spelling, and overall style by one or more of the highly qualified native English speaking editors at SNAS.

This certificate was issued on **March 5, 2024** and may be verified on the SNAS website using the verification code **074F-5CC2-42F2-E3F6-DB82**.

Neither the research content nor the authors' intentions were altered in any way during the editing process. Documents receiving this certification should be English-ready for publication; however, the author has the ability to accept or reject our suggestions and changes. To verify the final SNAS edited version, please visit our verification page at secure.authorservices.springernature.com/certificate/verify.
If you have any questions or concerns about this edited document, please contact SNAS at support@as.springernature.com.

SNAS provides a range of editing, translation, and manuscript services for researchers and publishers around the world.
For more information about our company, services, and partner discounts, please visit authorservices.springernature.com.

According to the publication requirements of scientific reports for electrophoretic gels and imprints: the figure legend must state that the samples derive from the same experiment and that gels/blots were processed in parallel.

To ensure that our banding was performed and obtained in the same wb experiment, we consider the close molecular weights of some proteins, e.g. Fkbp5 protein molecular weight is 51kda and actin protein molecular weight is 42kda； BAX protein molecular weight is 21kda and BCL2 protein molecular weight is 26kda. Therefore, when the same imprinted protein is transferred to the PVDF membrane, only a PVDF membrane with a width of 1.0cm can be prepared in advance to ensure that all PVDF bands are transferred and tested in the same Wb experiment. This ensures that all the figure legends come from the same Wb and makes our results more scientific. Therefore, the bands we currently provide are the original bands that were designed and prepared before the Wb experiment began. We hope the editor can understand and support our research. Thank you.

Figure 1C

Calnexin

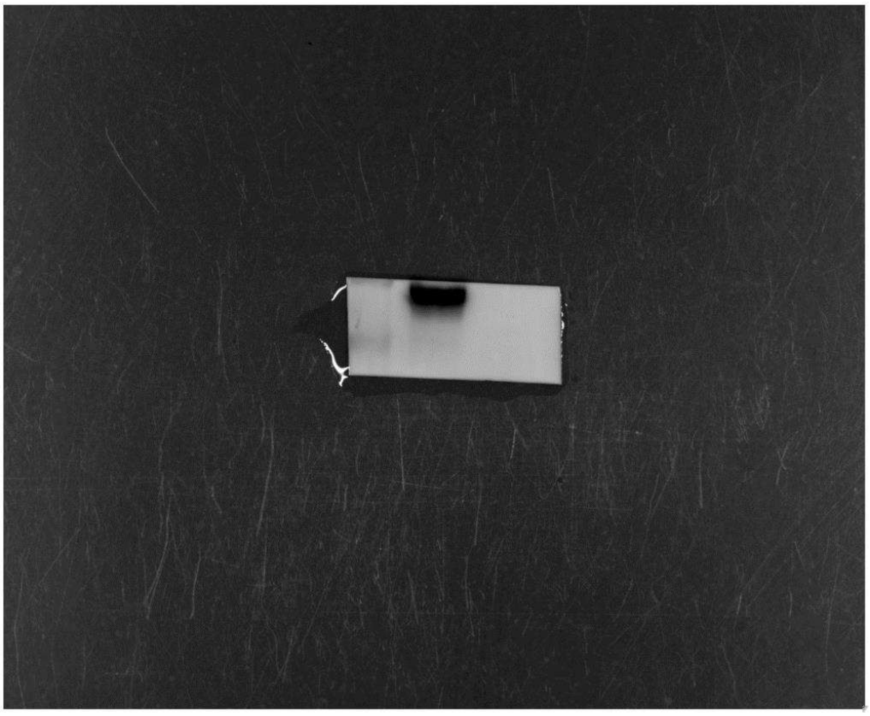

CD9

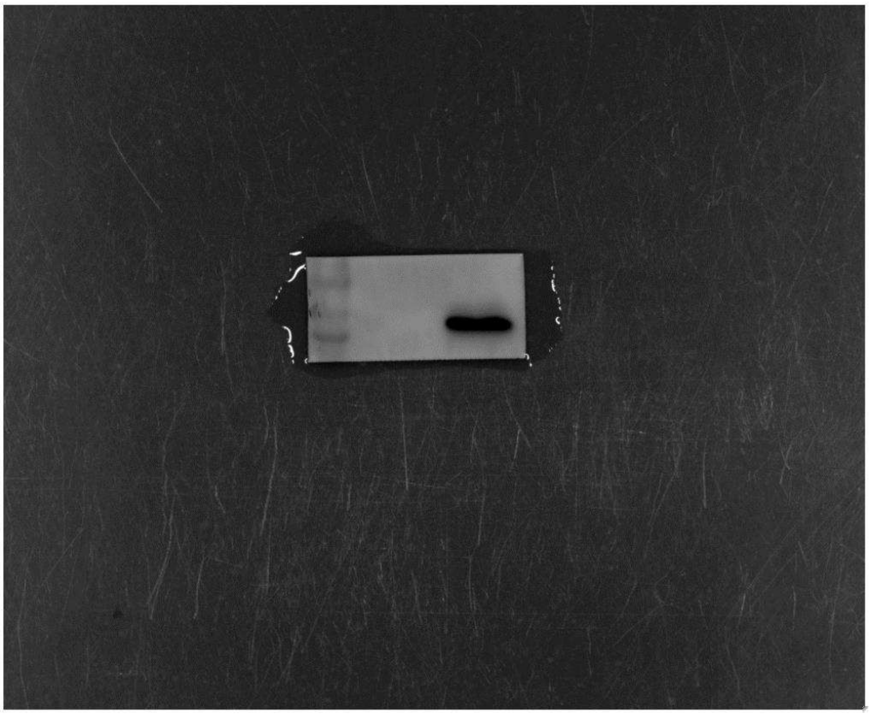

TSG101

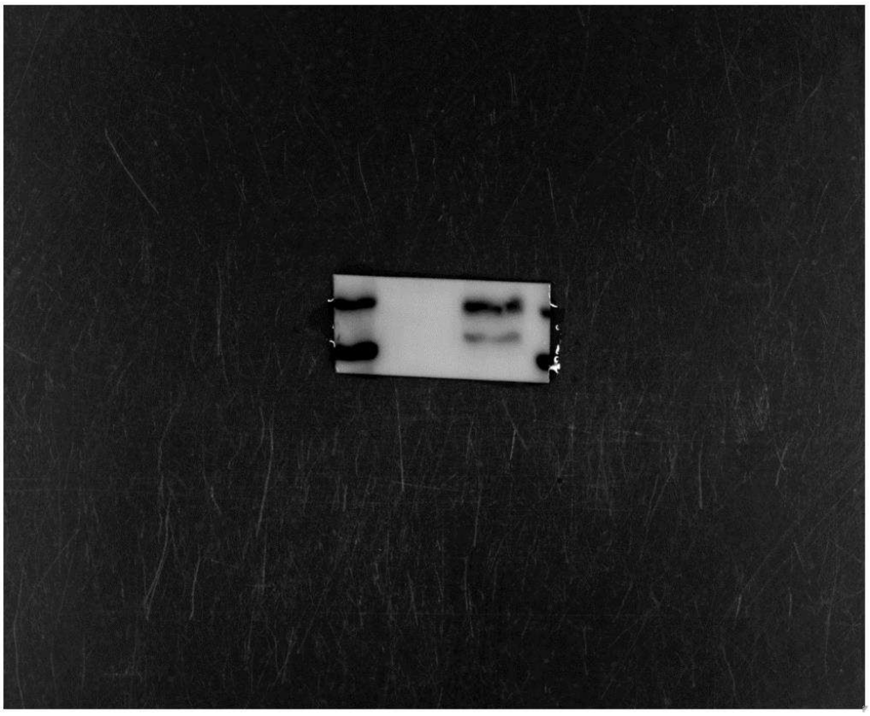

Figure 2C

BAX

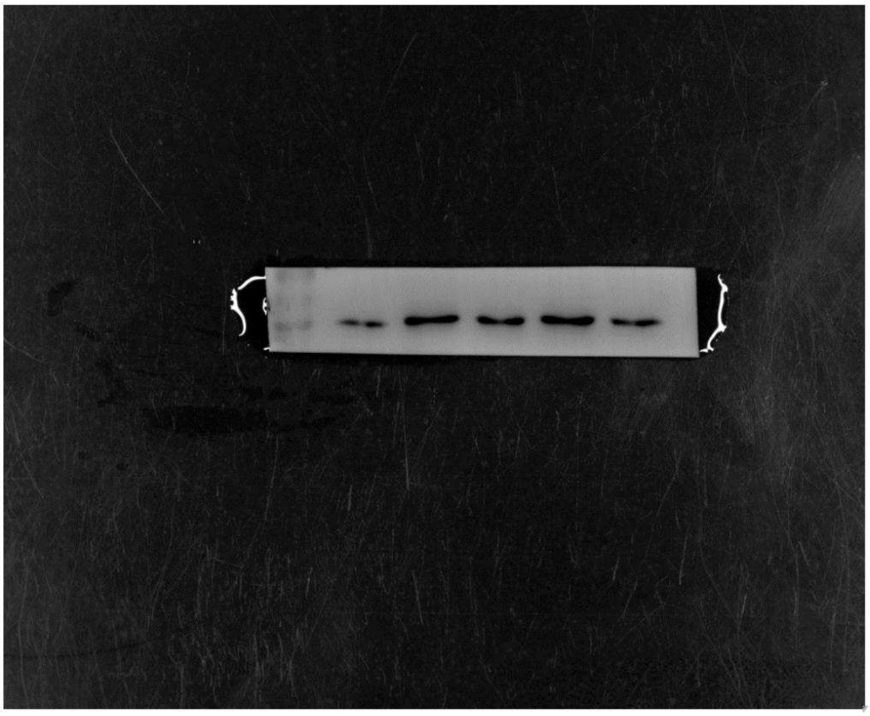

BCL2

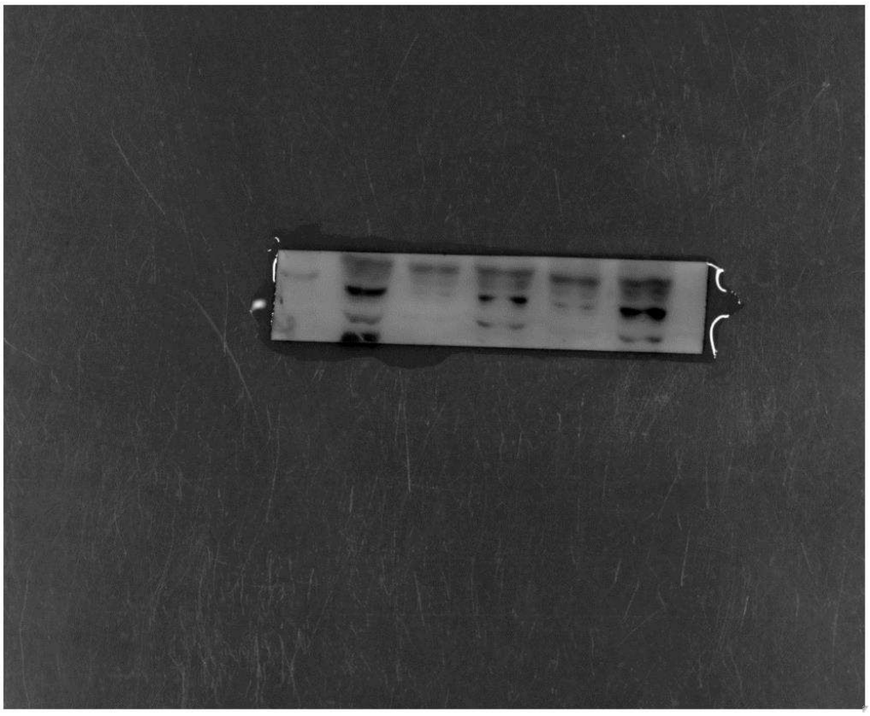

CC3

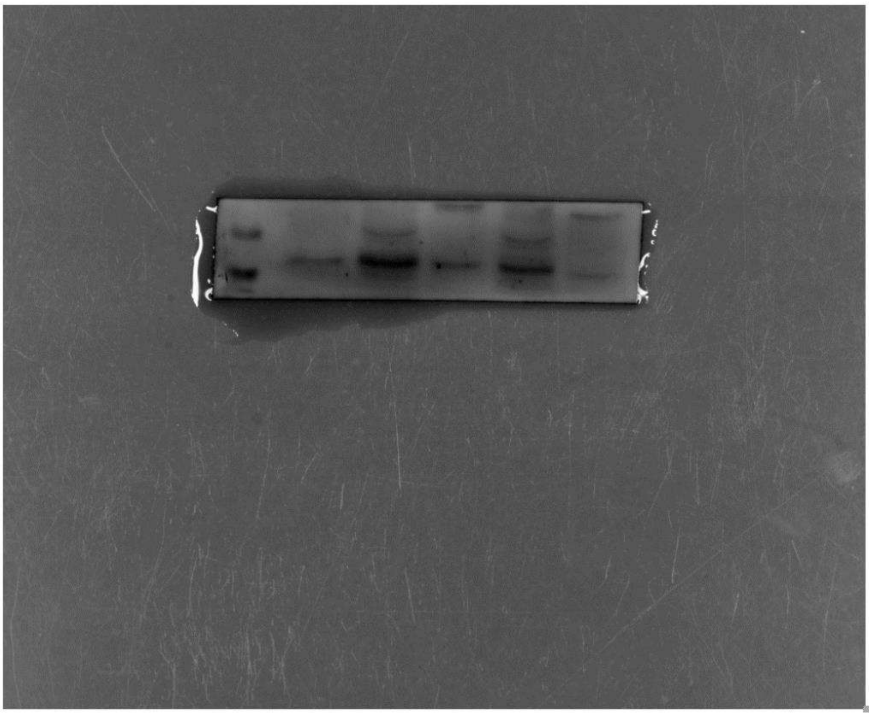

β-actin

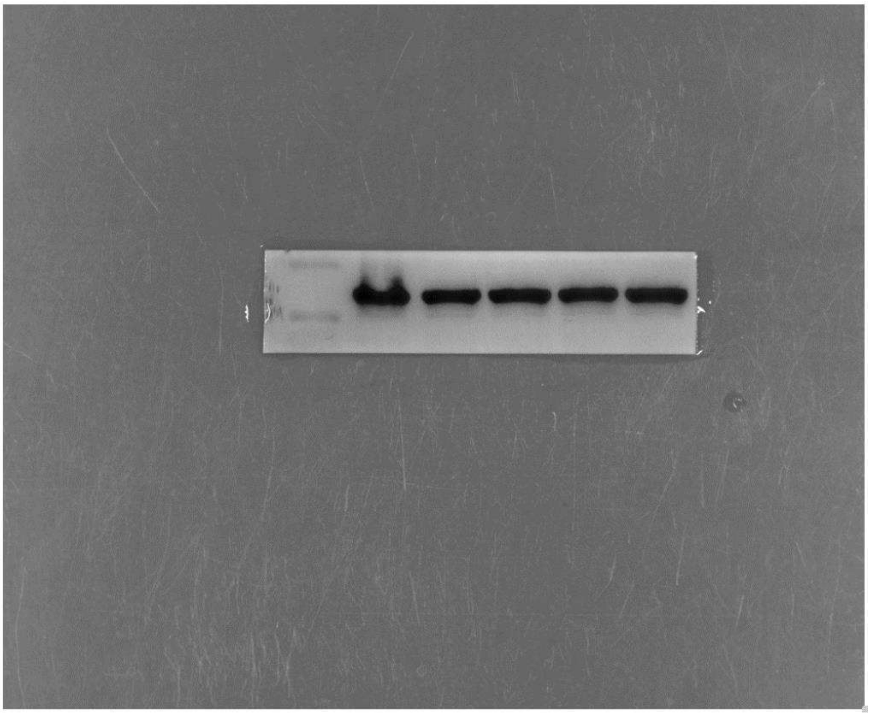

Figure 3E

BAX

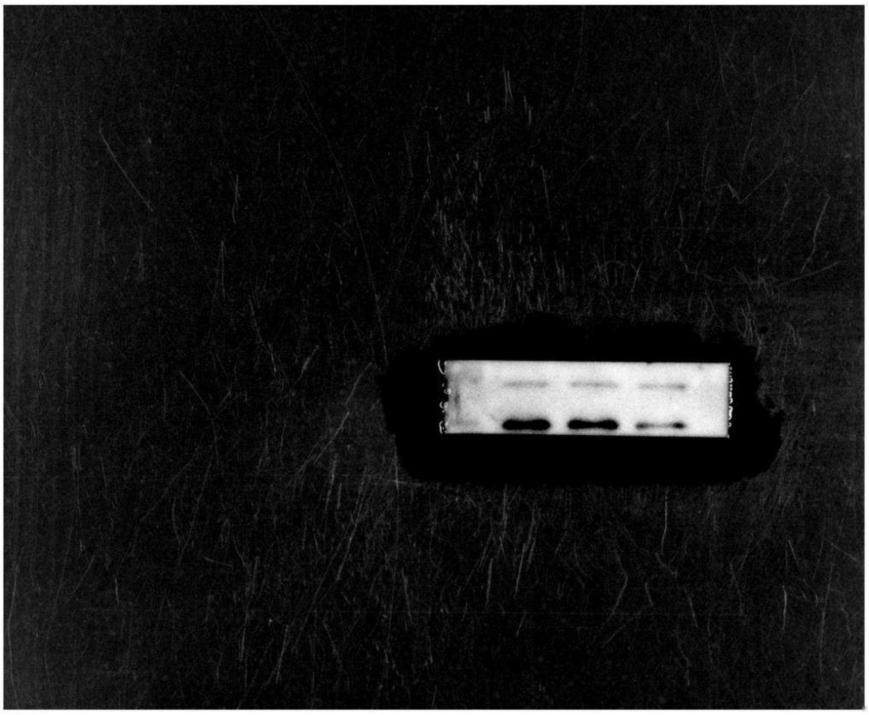

BCL2

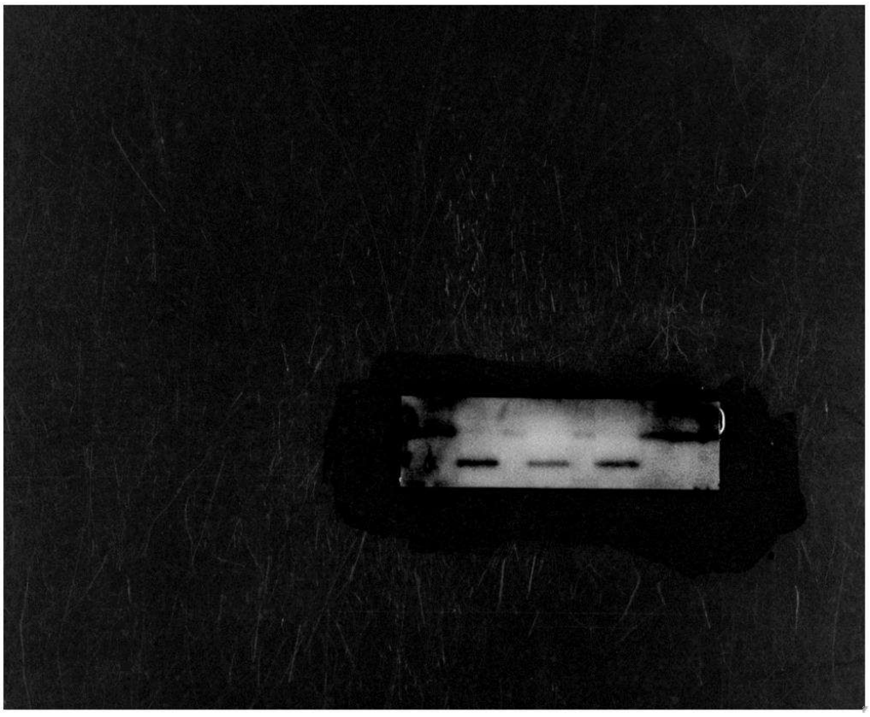

CC3

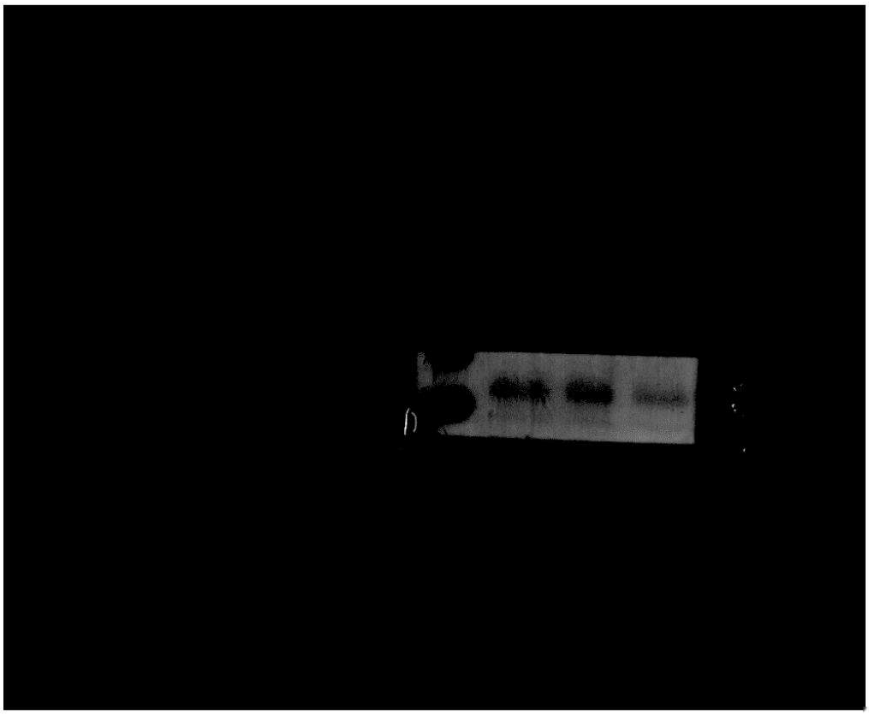

β-actin

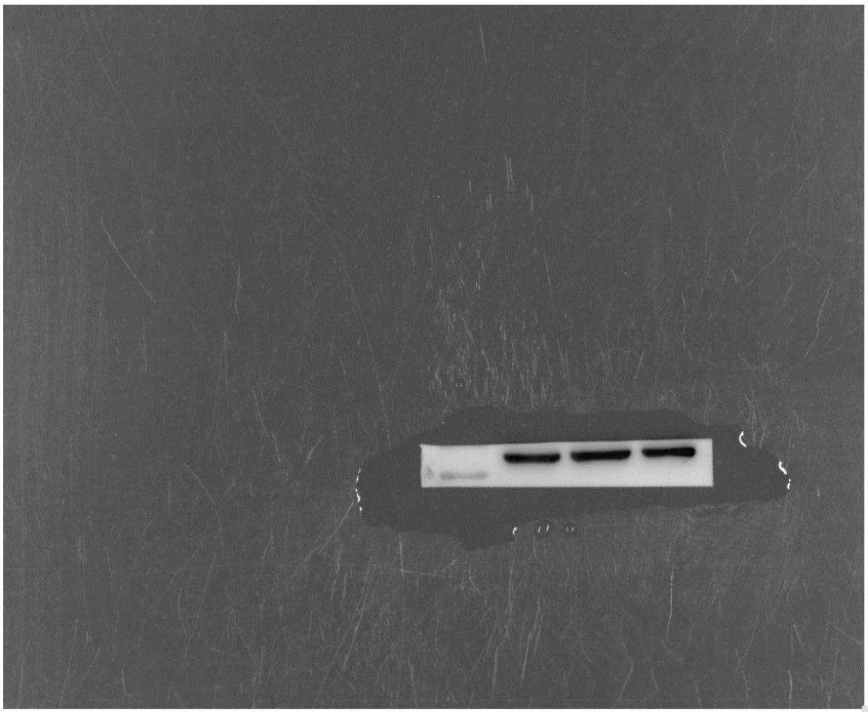

Figure5C

BCL2

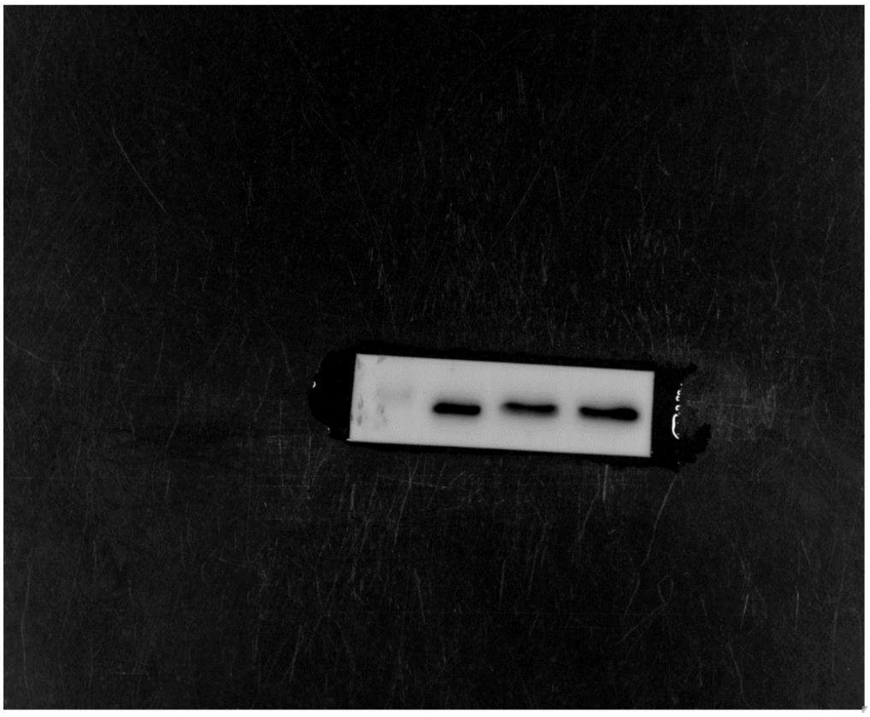

BAX

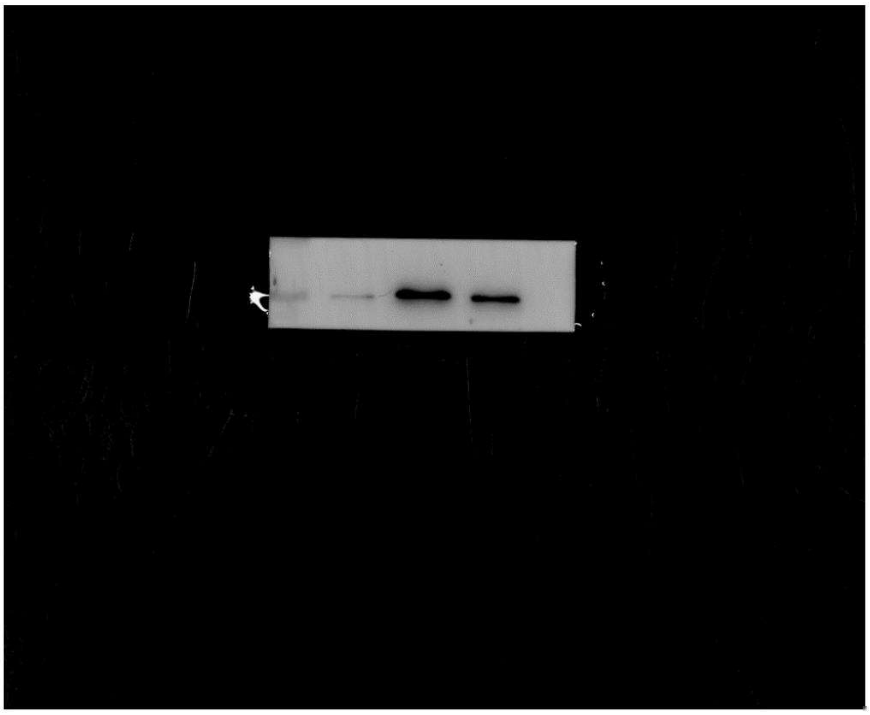

CC3

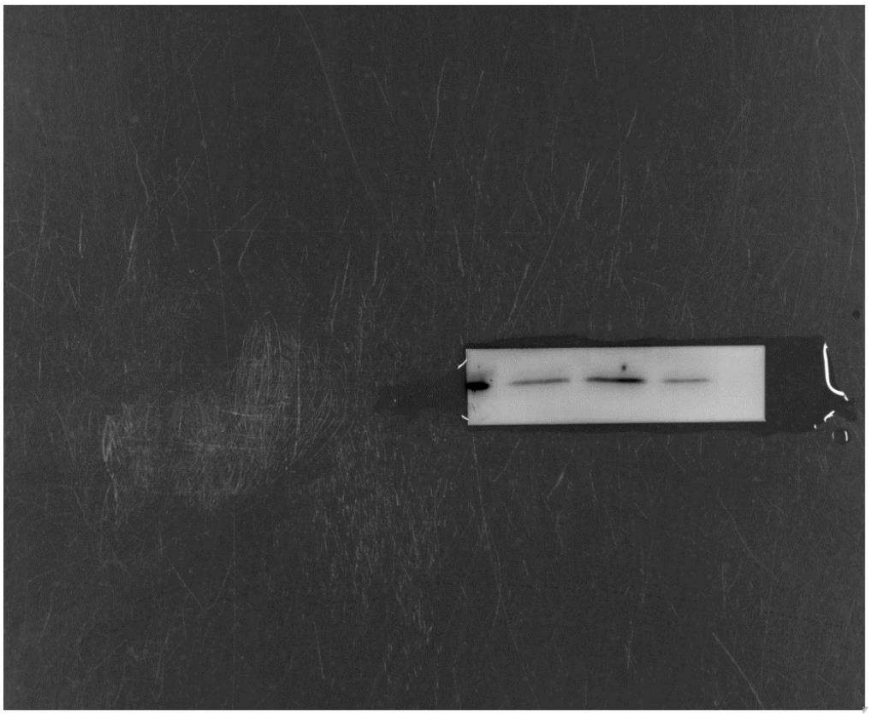

FKBP5

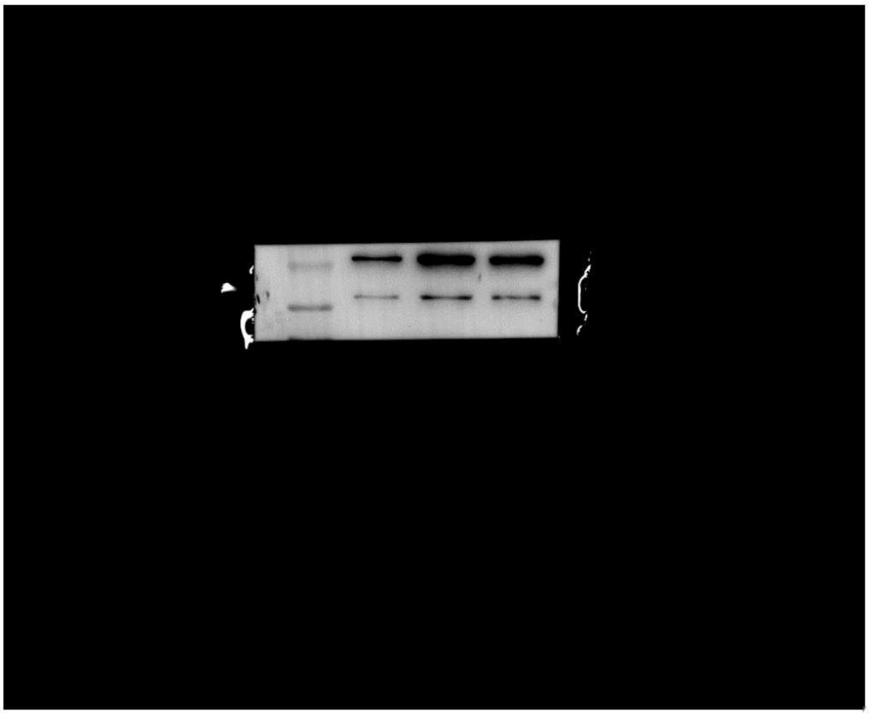

AKT

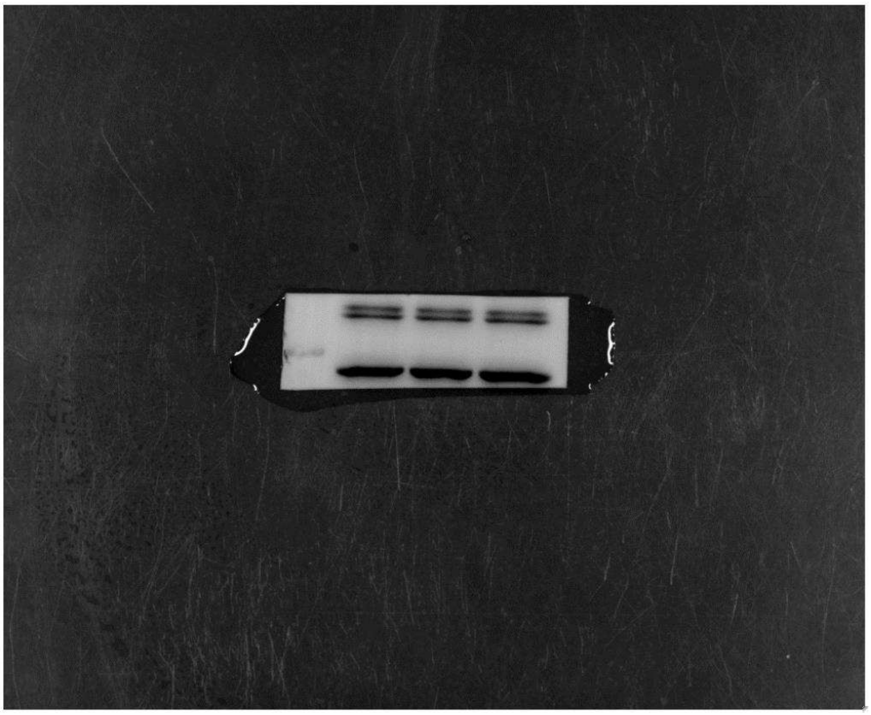

p-AKT

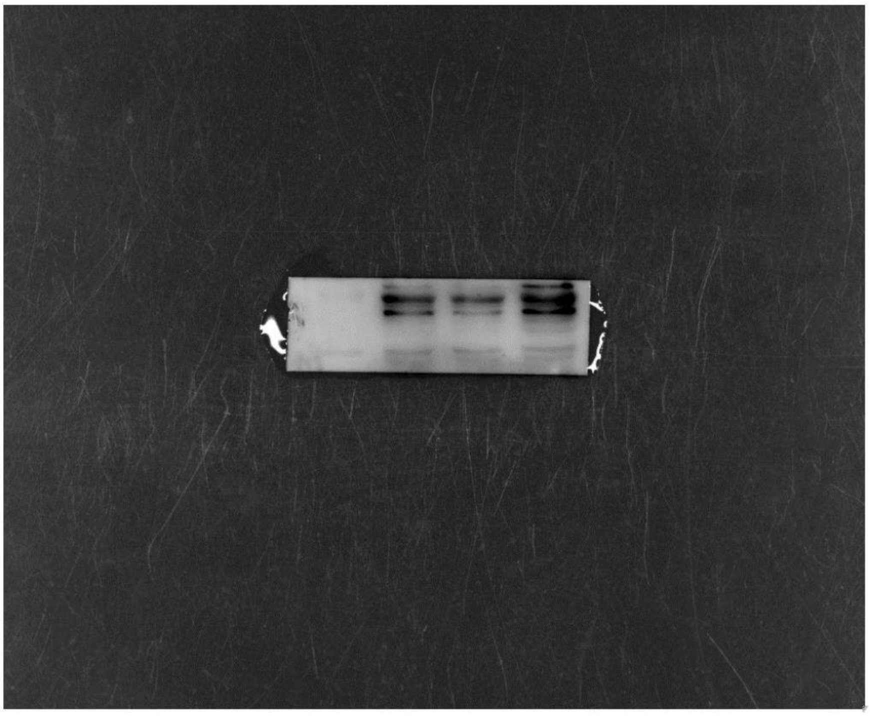

β-actin

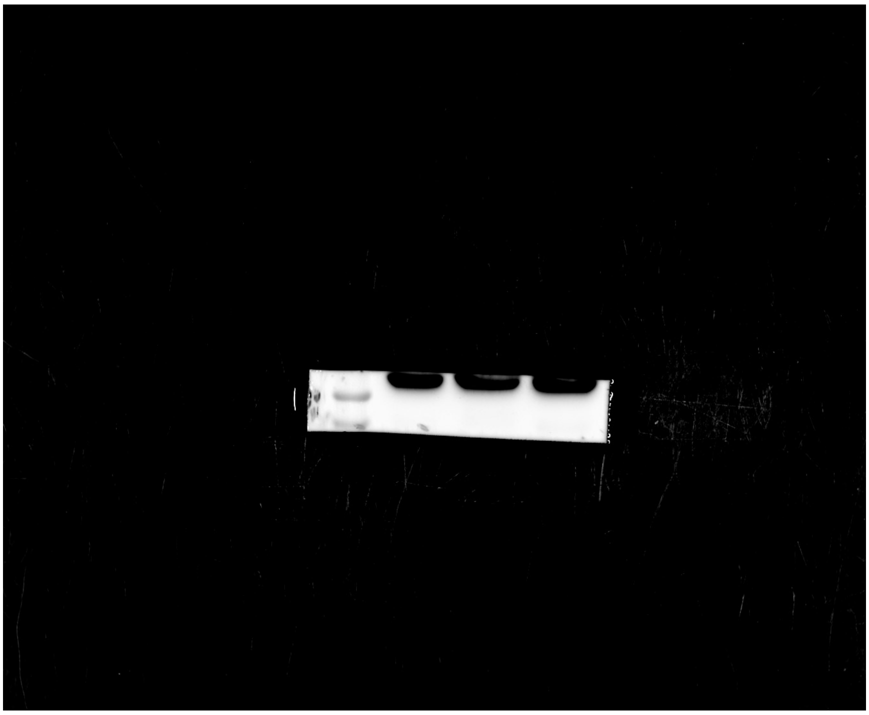

Figure 5E

FKBP5

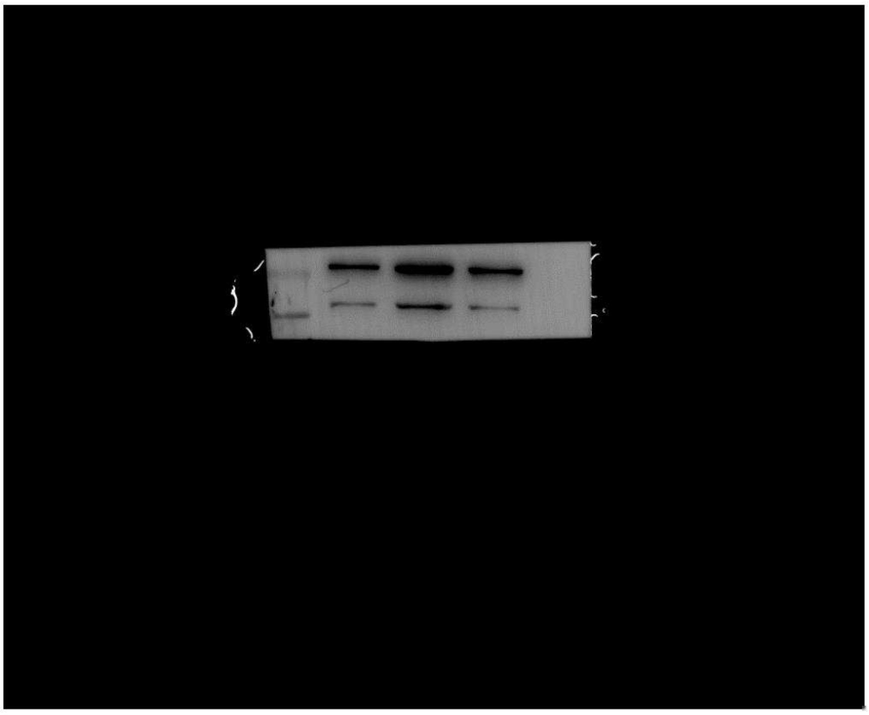

AKT

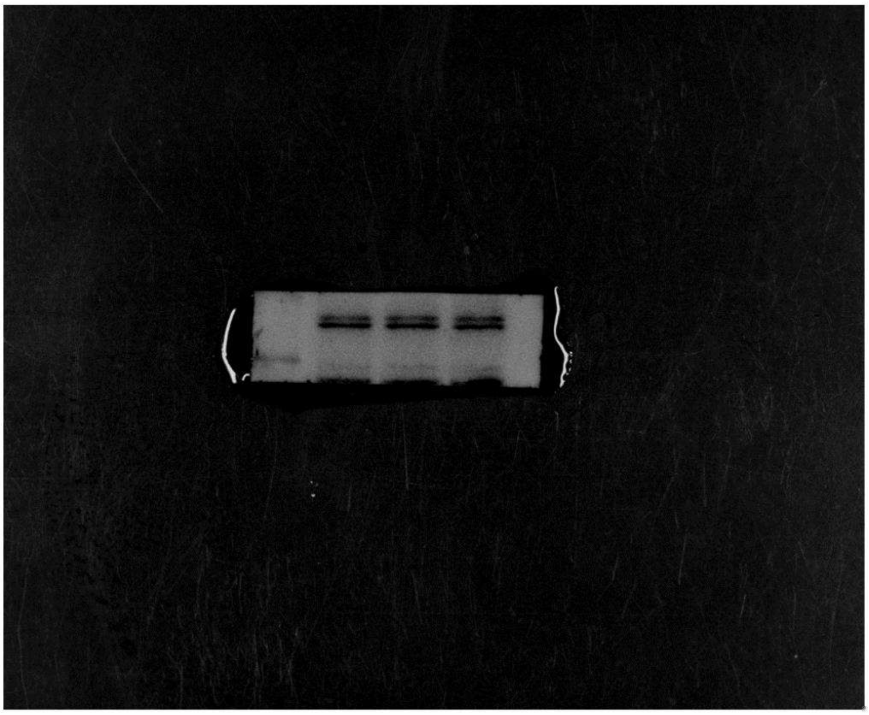

p-AKT

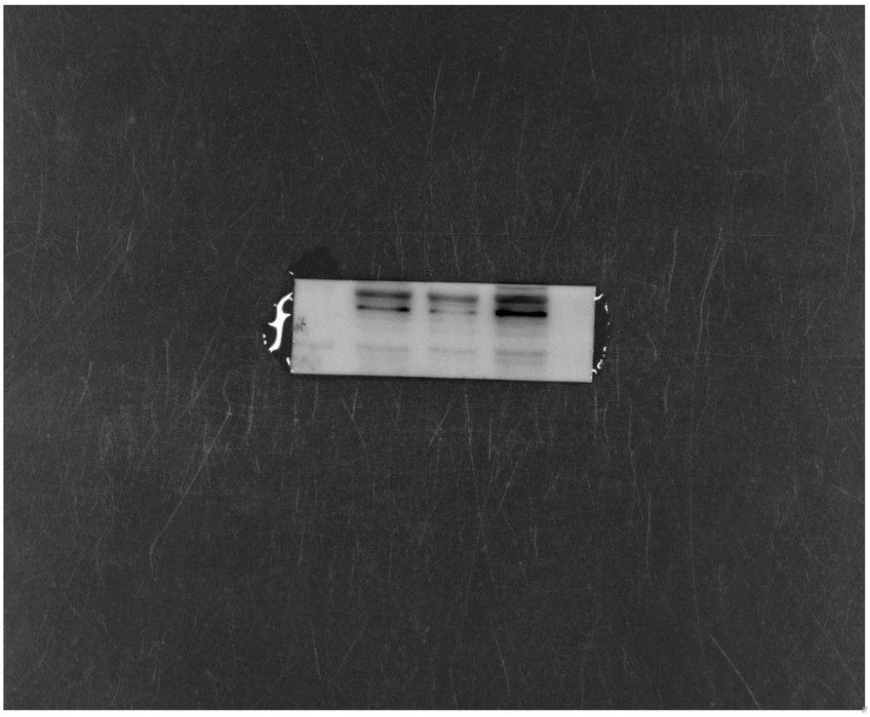

β-actin

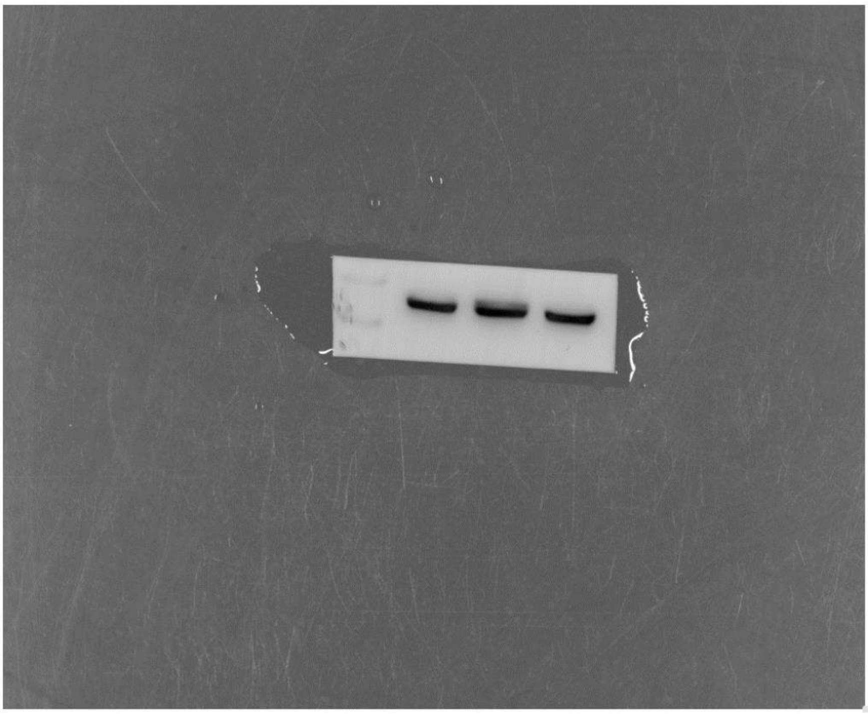

Figure 5H

FKBP5

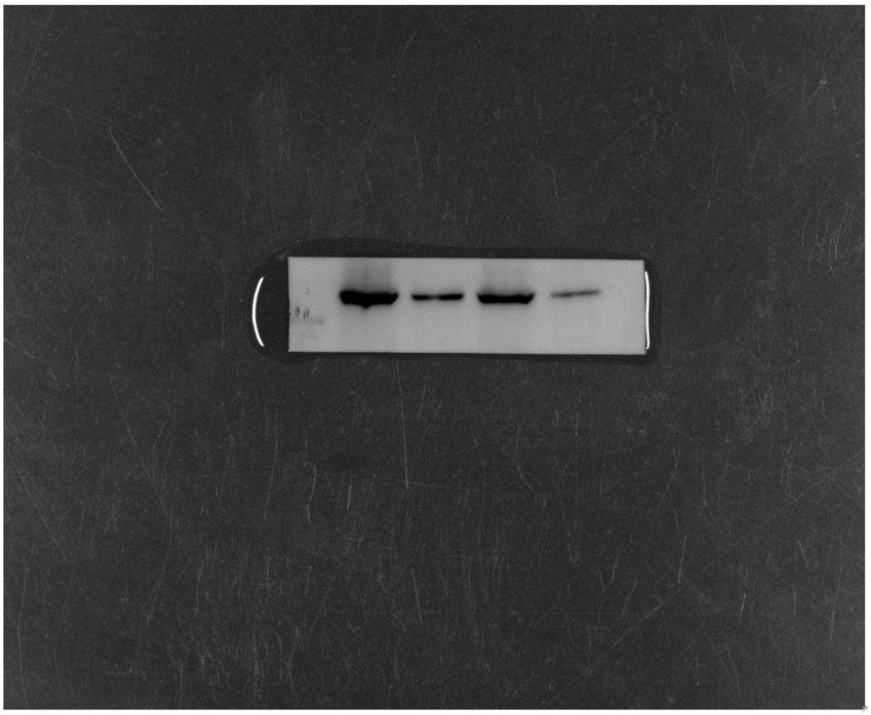

p-AKT

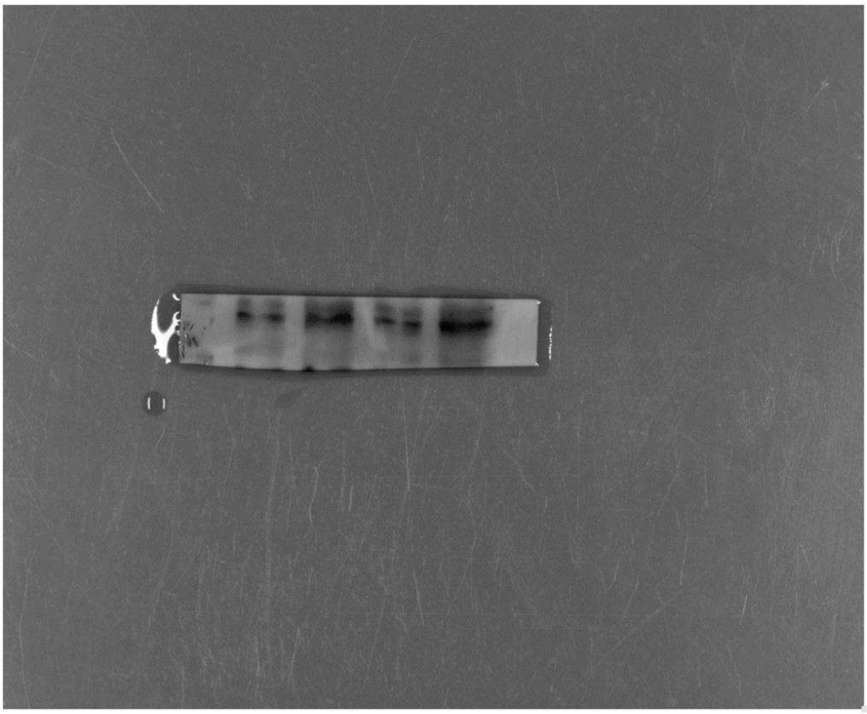

AKT

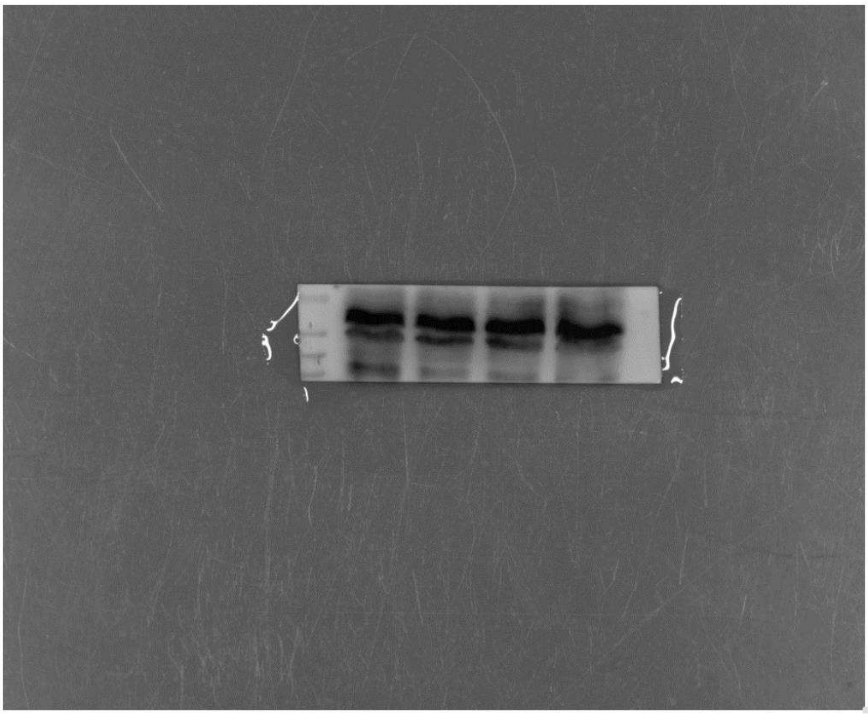

β-actin

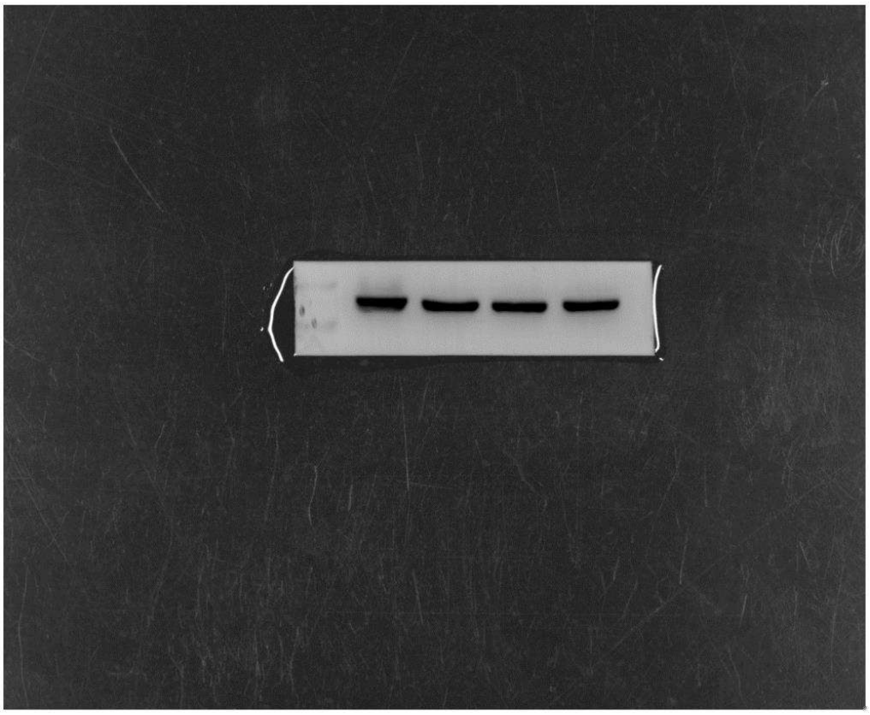

Supplement: Supplementary file 1 — Supplementary Information. [file 41598_2024_56950_MOESM1_ESM.pdf]
